# KIF11 As a Potential Pan-Cancer Immunological Biomarker Encompassing the Disease Staging, Prognoses, Tumor Microenvironment, and Therapeutic Responses

**DOI:** 10.1155/2022/2764940

**Published:** 2022-12-16

**Authors:** Xiuhong Guo, Li Zhou, Yuening Wu, Jingxiang Li

**Affiliations:** ^1^Luzhou Key Laboratory of Oral and Maxillofacial Reconstruction and Regeneration, The Affiliated Stomatological Hospital of Southwest Medical University, Luzhou 646000, China; ^2^State Key Laboratory of Biotherapy, West China Hospital of Sichuan University and Collaborative Innovation Center of Biotherapy, Chengdu 610041, China

## Abstract

KIF11 is one of the 45 family members of kinesin superfamily proteins that functions as a motor protein in mitosis. Emerging evidence revealed that KIF11 plays pivotal roles in cancer initiation, development, and progression. However, the prognostic, oncological, and immunological values of KIF11 have not been comprehensively explored in pan-cancer. In present study, we comprehensively interrogated the role of KIF11 in tumor progression, tumor stemness, genomic heterogeneity, tumor immune infiltration, immune evasion, therapy response, and prognosis of cohorts from various cancer types. In general, KIF11 was significantly upregulated in tumors compared with paired normal tissues. KIF11 showed strong relationships with pathological stage, prognosis, tumor stemness, genomic heterogeneity, neoantigens, ESTIMATE, immune checkpoint, and drug sensitivity. The methylation level of KIF11 decreased in most cancers and was correlated with the survival probability in different human cancers. The expression of KIF11 was diverse in different molecular and immune subtypes and remarkably correlated with immune cell infiltration in the tumor microenvironment. Comparative study revealed that KIF11 was a powerful biomarker and associated with immune, targeted, and chemotherapeutic outcomes in various cancers. In addition, KIF11 interaction and coexpression networks mainly participated in the regulation of cell cycle, cell division, p53 signaling pathway, DNA repair and recombination, chromatin organization, antigen processing and presentation, and drug resistance. Our pan-cancer analysis provides a comprehensive understanding of the functions of KIF11 in oncogenesis, progression, and therapy in different cancers. KIF11 may serve as a potential prognostic and immunological pan-cancer biomarker. Moreover, KIF11 could be a novel target for tumor immunotherapy.

## 1. Introduction

Nowadays, cancer has become a leading cause of death worldwide and forces a major health and economic burden on society [1]. Human carcinogenesis is a dynamic process that is regulated at multiple spatial and temporal scales [2]. The unique gene expression profile resulting from DNA changes including deletion, amplification, mutation, and translocation, and epigenetic alterations represents hallmarks of cancer development and provides a new perspective to understand the initiation and progression mechanism of tumor [3]. Pan-cancer analysis provides a powerful method to find common and distinctive characteristics of human cancers and provides novel ideas for the clinical therapy of tumors [4, 5].

Kinesin superfamily proteins (KIFs) are a group of proteins with a highly conserved motor domain that provides motors binding to microtubules [6, 7]. The motor ability of KIFs plays a vital role in mitosis, meiosis, vesicle and organelle trafficking, and the maintenance of cell polarity [8–10]. KIFs were first isolated from squid tissue and were ubiquitous in all eukaryotes [11]. A total of 45 KIF genes with various functions have been defined in human [12]. The KIFs are classified into 15 kinesin families termed kinesin-1 to kinesin-14B based on the phylogenetic relationship. These families can be broadly grouped into three types according to the position of the motor domain in the molecule: N kinesins have a motor domain in the amino terminal region, M kinesins have a motor domain in the middle region, and C kinesins have a motor domain in the carboxy terminal region [13]. In general, N kinesins provide microtubule-plus-end-directed motilities; M kinesins depolymerize microtubules into tubulin molecules, and C kinesins provide microtubule-minus-end-directed motilities [13–15]. The energy released by ATP hydrolysis in the motor domain provides force production for directional movement of KIFs [16]. In the past few years, numerous studies have showed that aberrant expression of KIFs is involved in the development and progress of different kinds of human cancers [17–19].

KIF11 also known as EG5, the unique member of kinesin-5 subfamily, is responsible for separating duplicated poles and maintaining proper spindle bipolarity during mitosis [20–22], secretory protein transport in nonmitotic cells [23], and regulating axonal growth and branching in developing neurons [24, 25]. KIF11 is a member of the N kinesins that contain a motor domain in the amino terminal region of the molecule. An intact KIF11 protein is composed of a motor domain, a neck linker domain, and a tail domain assembling as an antiparallel tetrameric structure, which allows them to bundle and slide parallel and antiparallel microtubules [20, 26, 27]. KIF11 generally moves slower and is less processive than conventional transport kinesins [20, 28]. In recent years, numerous studies have shown that KIF11 participates in the growth and development of a variety of human cancers [29]. Due to its crucial mitotic function, KIF11 is a target for potential anticancer drugs, emphasizing the importance of a more thorough understanding of its cellular functions.

**Figure 1:**
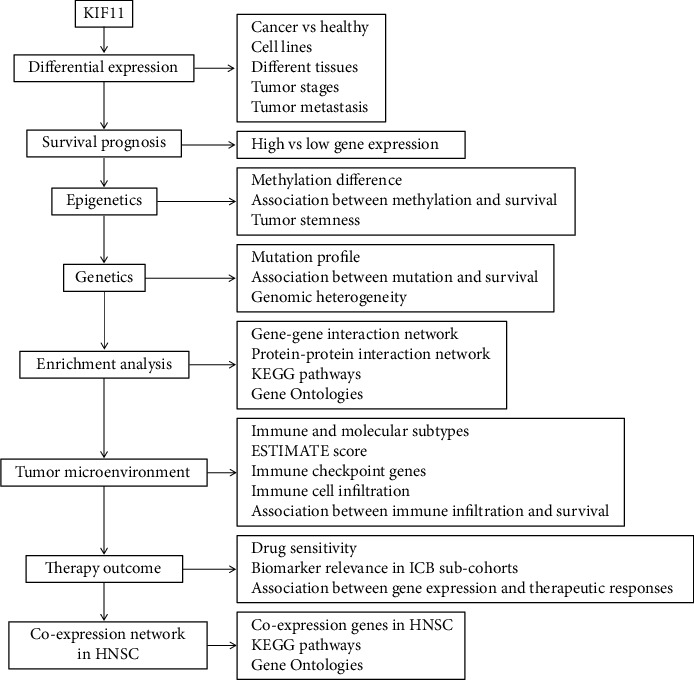
The flow chart of the study.

Recent studies have revealed that KIF11 plays an important role in cancer. However, the function of KIF11 in tumorigenesis and tumor progression remains largely unknown from the perspective across multiple cancers. In the present study, we conducted a comprehensive analysis of the KIF11 gene based on multiomics data to investigate the roles of KIF11 in oncogenesis, progression, and therapy from the perspective of pan-cancer. We analyzed KIF11 expression in pan-cancer, normal tissues, and various cell lines and evaluated the prognostic value and biomarker relevance of KIF11 in different human cancers. Furthermore, the potential associations between KIF11 expression and molecular subtypes, immune subtypes, neoantigen, ESTIMATE, immune checkpoint, and immune cell infiltration in the tumor microenvironment were analyzed. In addition, we explored the relationships between KIF11 and tumor stemness, genomic heterogeneity, drug sensitivity, and therapy response in human cancers. The interaction and coexpression networks of KIF11 were constructed to evaluate KIF11 associated pathways. This study would provide insights into the role of KIF11 in cancer initiation, progression, and tumor immunotherapy from the perspective of pan-cancer, but some limitations still exist. First, although we investigated the protein level of KIF11 via the IHC data of HPA database, the IHC results of some cancer types are missing in the HPA database. Second, we observed that KIF11 was correlated with immune cell infiltration in the tumor microenvironment. However, the molecular mechanisms and roles of KIF11 in immune infiltration and escape need to be explored in further studies. Third, most of the analyses were performed based on multiomics data, the precise verification and high-quality evidence should be further performed and provided by clinical trials and biological experiments. The workflow of this study is shown in Figure 1.

## 2. Materials and Methods

### 2.1. Gene Expression Analysis

The TIMER database (https://cistrome.shinyapps.io/timer/), GEPIA database (http://gepia2.cancer-pku.cn/#analysis), and UALCAN database (http://ualcan.path.uab.edu/index.html) were used to compare KIF11 expression between human tumors and normal tissues. The HPA database (https://www.proteinatlas.org/), SangerBox website (http://sangerbox.com/Tool), and GeneCards database (https://www.genecards.org/) were used to analyze the expression profiles of KIF11 in different normal tissues and cell lines. The HPA database was explored to validate the KIF11 protein expression in human cancers by immunohistochemical staining with anti-KIF11 antibody (Atlas Antibodies, Cat#HPA006916, RRID: AB_1848033). The detailed information of cancer tissue material and clinicopathological data used in this study was listed in Supplementary Table 2. The IHC staining protocol can be found at https://www.proteinatlas.org/download/IHC_protocol.pdf. The detailed information of the cell lines in the HPA database can be found at https://www.proteinatlas.org/learn/cellines. As for HPA database, mRNA sequencing was performed with a read length of 2 × 100 bases, producing an average of 18 million mappable read pairs per sample. For GTEx database, RNA sequencing was performed using a 76 base, paired-end Illumina TruSeq RNA protocol, averaging ~50 million aligned reads per sample. For TCGA database, RNA-seq data are of high quality with a mean coverage of around 50 fold. For TARGET database, approximately 21500 genes were covered by at least one read, and about 12990 genes had RPKM (Reads Per Kilobase per Million) mapped reads with values ≥ 1. The methods description partly reproduces the wording of the database.

### 2.2. Prognostic Analysis

The SangerBox website, GEPIA database, Kaplan-Meier's plotter database (http://kmplot.com/analysis/), and PrognoScan databases (http://dna00.bio.kyutech.ac.jp/PrognoScan/index.html) were used to exam the connection between KIF11 expression and the prognosis of patients, including overall survival (OS), disease free interval (DFI), disease specific survival (DSS), and progression free interval (PFI). Data of the SangerBox website was collected from the TCGA and GTEx database. The log rank test was used for statistical analysis. For GEPIA database, the median KIF11 expression was used as a cutoff value to classify groups, and the hazards ratio was calculated based on Cox PH Model. The Kaplan-Meier plotter database splits patients by calculating an optimal cutoff value automatically.

### 2.3. Epigenetic Methylation Analysis

UALCAN database and DiseaseMeth database (http://bio-bigdata.hrbmu.edu.cn/diseasemeth/index.html) were used to compare the methylation status of KIF11 promoter between human tumors and normal tissues. The EWAS database (https://ngdc.cncb.ac.cn/ewas/datahub/exploration) and MethSurv database (https://biit.cs.ut.ee/methsurv/) were used to compare the methylation status of single CpG island in KIF11 promoter between tumor and normal tissue and to study the correlation between methylation status and survival probability in different human cancers. The connection between KIF11 and the cancer stemness was investigated via the SangerBox website using the data from UCSC database (https://xenabrowser.net/).

### 2.4. Genetic Alternation Analysis

The cBio Cancer Genomics Portal (c-BioPortal) (http://cbioportal.org) was applied to explore KIF11 genomic alterations in different human cancers. The connections between KIF11 expression and genomic heterogeneity and the mutation map of KIF11 were investigated via the SangerBox website using the data from UCSC database (https://xenabrowser.net/) and GDC database (https://portal.gdc.cancer.gov/), respectively.

### 2.5. Interaction Network, Gene Ontology (GO) Term, and Kyoto Encyclopedia of Genes and Genomes (KEGG) Pathway Enrichment Analyses

The GeneMANIA database (http://www.genemania.org) was applied to construct the gene-gene interaction network of KIF11. A total of 20 related genes were shown. The STRING database (https://string-db.org/) was used to construct the protein-protein interaction network of KIF11. The main parameters were set as follows: network type “full STRING network”, meaning of network edges “evidence”, active interaction sources “Experiments, Text mining, Databases”, and minimum required interaction score “medium confidence (0.400)”. Fifty KIF11 binding proteins were used for GO and KEGG analyses using the DAVID Bioinformatics Resources (https://david.ncifcrf.gov/). The top 10 enrichment pathways were displayed.

### 2.6. Immune and Molecular Subtypes Analysis

The TISIDB database (http://cis.hku.hk/TISIDB/index.php), which integrates multiple data types to assess tumor and immune system interaction, was used to explore the correlations between KIF11 expression and molecular or immune subtypes in different human cancers.

### 2.7. Coexpression Network Analysis

The LinkedOmics database (http://www.linkedomics.org/login.php) was used to explore the KIF11 coexpression genes in HNSC using RNAseq dataset by Spearman's correlation test. Top 50 positive or negative coexpression genes were displayed via heat map and volcano plot. Furthermore, gene ontology biological process and KEGG pathways of KIF11 and the coexpression genes were explored and displayed via bar chart and volcano plot.

### 2.8. Analysis of the Connections between KIF11 and Neoantigen, ESTIMATE, and Immune Checkpoint Genes

The connections between KIF11 expression and neoantigen, ESTIMATE, and immune checkpoint genes were investigated via the SangerBox website using Spearman's method. ESTIMATE is a common algorithm for predicting tumor purity, consisting of stromal score, immune score, and estimate score. Immune checkpoint genes including immune stimulators and immune inhibitors were selected according to the previous study [30].

### 2.9. Immune Cell Infiltration Analysis

The connections between KIF11 expression and the infiltration level of T cells, CD8^+^ T cells, cytotoxic lymphocytes, B lineage, natural killer cells, monocytic lineage, myeloid dendritic cells, neutrophils, endothelial cells, and fibroblasts were investigated via the SangerBox website by MCP-counter. The connections between KIF11 expression and the infiltration level of immunosuppressive cells including myeloid-derived suppressor cells (MDSCs), cancer-associated fibroblasts (CAFs), and regulatory T cells (Tregs) were investigated via the TIMER database through different methodologies, including TIDE, EPIC, CIBERSORT, and MCP counter. The Kaplan-Meier plotter database was used for prognosis analyses based on the expression level of KIF11 in related immune cell subgroups.

### 2.10. Drug Sensitivity and Therapeutic Response Analysis

The RNAactDrug database (http://bio-bigdata.hrbmu.edu.cn/RNAactDrug/), a comprehensive database of RNAs associated with drug sensitivity from multiomics data, was used to investigate the connections between drug sensitivity and KIF11 at three molecular levels (expression, copy number variation, and methylation). The TIDE server (http://tide.dfci.harvard.edu/), a module that can perform the comparison between the custom biomarker and other published biomarkers based on their predictive power of response outcome and overall survival, was used to compare the predictive power of KIF11 with nine standardized biomarkers of tumor immune response, including TIDE, MSI score, TMB, CD274, CD8, IFNG, T. Clonality, B. Clonality, and merck18. The ROC plotter server (https://www.rocplot.org/site/index) was applied to explore the association between KIF11 expression and therapeutic response in breast and ovarian cancer patients.

### 2.11. Statistical Analysis

R software and the attached packages were utilized for statistical analysis. ANOVA, Wilcoxon's test, Kruskal-Wallis' test, and *t* test were used in GEPIA, TIMER, SangerBox, and UALCAN database for differential expression analysis, respectively. Cox regression analysis and the Kaplan-Meier curve were used to analyze the association between KIF11 and patient survival. The *P* value obtained from log-rank test was used to indicate statistical significance. Spearman or Pearson's correlation method was used to calculate the correlation between two variables. Mann–Whitney test and Receiver Operating Characteristic test were used in ROC plotter server for cohorts comparison. Quantitative real-time PCR results are reported as the mean ± SD. Statistical significance between samples was determined by *t* test. Statistical significance was set at *P* < 0.05.

## 3. Results

### 3.1. KIF11 Expression Analysis in Pan-Cancer

**Figure 2:**
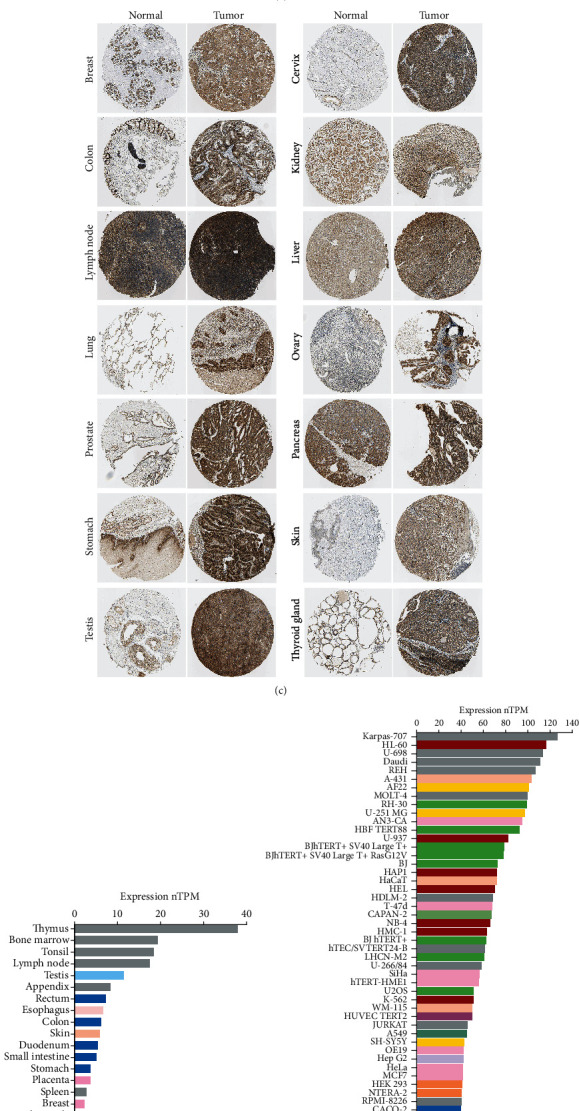
The expression level of KIF11 across different cancers. (a) KIF11 expression level in different cancer types and normal tissues analyzed by the TIMER database. ^∗^*P* < 0.05, ^∗∗^*P* < 0.01, ^∗∗∗^*P* < 0.001. (b) KIF11 expression level in different cancer types and normal tissues analyzed by the GEPIA database. (c) Immunohistochemical analysis of KIF11 in different tumors and normal tissues. Each sample is represented by 1 mm tissue cores. (d) KIF11 expression level in different normal tissues analyzed by the HPA database. (e) KIF11 expression level in different cell lines analyzed by the HPA database.

According to the results from the TIMER database, KIF11 mRNA level was significantly higher in most tumors versus adjacent normal tissues, such as BLCA, BRCA, CHOL, COAD, ESCA, HNSC, KIRC, LIHC, LUAD, LUSC, PRAD, READ, STAD, and UCEC (Figure 2(a)). The results from the GEPIA database also showed that KIF11 expression was significantly higher in most human cancers including ACC, BLCA, BRCA, CESC, CHOL, COAD, DLBC, ESCA, GBM, LGG, LUAD, LUSC, OV, PAAD, READ, SARC, SKCM, STAD, THYM, UCEC, and UCS (Figure 2(b)). The results of the UALCAN database showed that KIF11 expression was significantly higher among most cancer types, which was consistent with the TIMER and GEPIA database results (Supplementary Figure 1A). Moreover, immunohistochemical staining for KIF11 was investigated via the HPA database. The expressing level of KIF11 was higher in most tumors compared with normal tissues (Figure 2(c)).

Additionally, we investigated the mRNA expression level of KIF11 across different normal tissues and cancer cell lines via the HPA database. KIF11 was lower expressed in most normal tissues, and higher expression was found in thymus, bone marrow, tonsil, and lymph node, all of which were associated with immune responses (Figure 2(d)). In contrast, KIF11 was high expressed in almost all cancer cell lines (Figure 2(e)). The results from the GTEx and CCLE database also proved that KIF11 mRNA expression level was low among most normal tissues except bone marrow and testis but high in almost all cancer cell lines (Supplementary Figures 1B, 1C). Further comparison of the KIF11 protein expression according to the GeneCards database demonstrated that KIF11 protein expressed at a low level in most normal tissues but high expressed in ovary and testis (Supplementary Figure 1D). In contrast, KIF11 protein expressed at a high level in almost all cancer cell lines, which was consistent with the HPA, GTEx, and CCLE database results (Supplementary Figure 1E). These results together suggested that KIF11 expression was abnormally upregulated in various cancers.

### 3.2. The Relationship between KIF11 Expression and Different Clinical Characteristics

**Figure 3:**
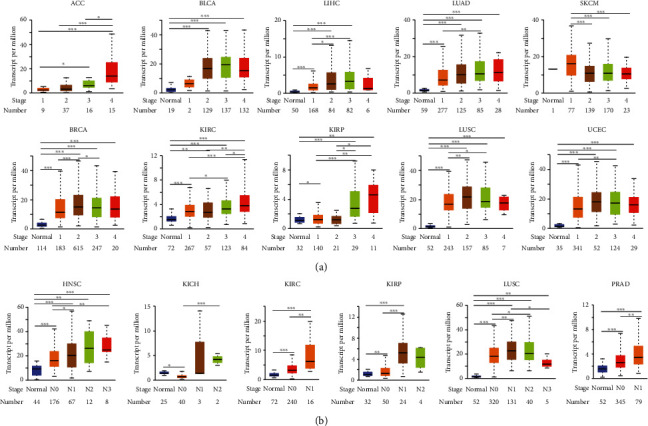
The expression level of KIF11 in different clinical characteristics. (a) KIF11 expression level in different cancer stages. (b) KIF11 expression level in different nodal metastasis status.

The KIF11 expression among groups of patients according to different clinical parameters was investigated by the UALCAN online tool. KIF11 was differentially expressed in different cancer stages, nodal metastasis status, and TP53 mutation status. According to pathological stages, KIF11 expression showed a trend variation along with the disease progression in ACC, BLCA, LIHC, LUAD, SKCM, BRCA, KIRC, KIRP, LUSC, and UCEC (Figure 3(a)). Regarding nodal metastasis status, a gradient increase of KIF11 expression was observed along with the progression of nodal metastasis in patients with HNSC, KIRC, KIRP, LUSC, and PRAD (Figure 3(b)). KIF11 expression was upregulated in both TP53 wild-type and TP53-mutant cancer patients compared to normal controls. In addition, KIF11 expression was higher in TP53-mutant cancer than TP53 wild-type cancer in most cancer types (Supplementary Figure 2). These results suggested that KIF11 expression was closely correlated with tumor progression and metastasis.

### 3.3. Prognostic Value of KIF11 in Pan-Cancer

**Figure 4:**
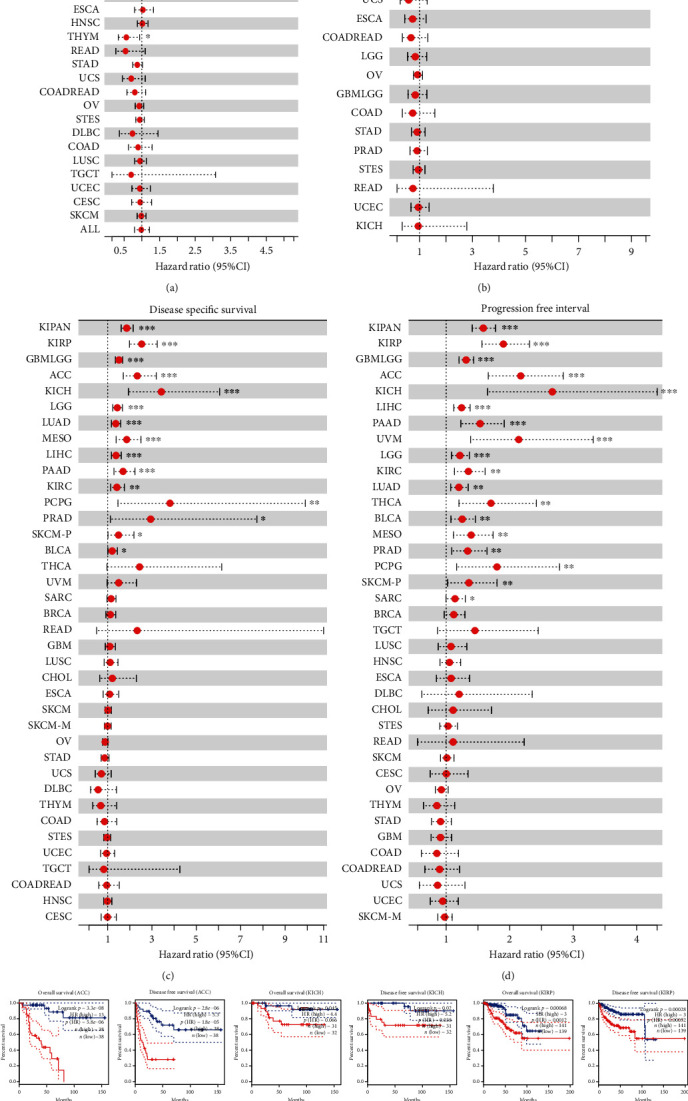
The prognostic value of KIF11 in human cancers. (a) Forest map shows the univariate cox regression results of KIF11 for OS. (b) Forest map shows the univariate Cox regression results of KIF11 for DFI. (c) Forest map shows the univariate Cox regression results of KIF11 for DSS. (d) Forest map shows the univariate Cox regression results of KIF11 for PFI. (e) The Kaplan-Meier survival curve of human cancers with high and low KIF11 expression analyzed by the GEPIA database.

To better understand the prognostic value and potential mechanism of KIF11 expression in pan-cancer, we comprehensively analyzed the prognostic value of KIF11 in human cancers by Cox proportional hazards model. Cox regression analyses of the correlations between KIF11 and OS, DFI, DSS, and PFI in different cancers were displayed in forest chart. Highly expressed KIF11 correlated negatively with OS in KIPAN, KIRP, ACC, LGG, KICH, LIHC, MESO, LUAD, PAAD, LAML, KIRC, PCPG, and PRAD and positively with OS in THYM (Figure 4(a)), negatively with DFI in KIRP, KIPAN, THCA, PAAD, LIHC, and SARC (Figure 4(b)), negatively with DSS in KIPAN, KIRP, GBMLGG, ACC, KICH, LGG, LUAD, MESO, LIHC, PAAD, KIRC, PCPG, PRAD, SKCM-P, and BLCA (Figure 4(c)), and negatively with PFI in KIPAN, KIRP, GBMLGG, ACC, KICH, LIHC, PAAD, UVM, LGG, KIRC, LUAD, THCA, BLCA, MESO, PRAD, PCPG, SKCM-P, and SARC (Figure 4(d)).

For further identification of the prognostic significance of KIF11 gene, the Kaplan-Meier survival curve of human cancers with high or low KIF11 expression was analyzed by the GEPIA database. Higher level of KIF11 mRNA indicated worse OS or DFS in ACC, KICH, KIRP, LGG, LIHC, LUAD, MESO, PAAD, SARC, and UVM, while opposite result was observed in THYM (Figure 4(e)). In the Kaplan-Meier plotter database, higher KIF11 expression was associated with poorer OS in KIRP, LIHC, LUAD, PAAD, PCPG, SARC, KIRC, UCES, and ESCA and DFS in KIRP, LIHC, LUAD, PAAD, PCPG, SARC and THCA. In contrast, higher KIF11 expression was related to better OS for patients with STAD, CESC, THYM, and DFS for patients with ESCA (Supplementary Figure 3). Furthermore, the correlation between KIF11 expression and survival was evaluated by PrognoScan database. Higher expression of KIF11 showed worse survival in bladder, brain, breast, eye, lung, ovarian, prostate, renal cell, skin, and soft tissue cancers (Supplementary Table 3). All the results above indicated that KIF11 expression was closely related to the prognosis of various cancer types.

### 3.4. KIF11 Correlated with Cancer Stemness and Showed Characteristic Epigenetic Modification in Pan-Cancer

**Figure 5:**
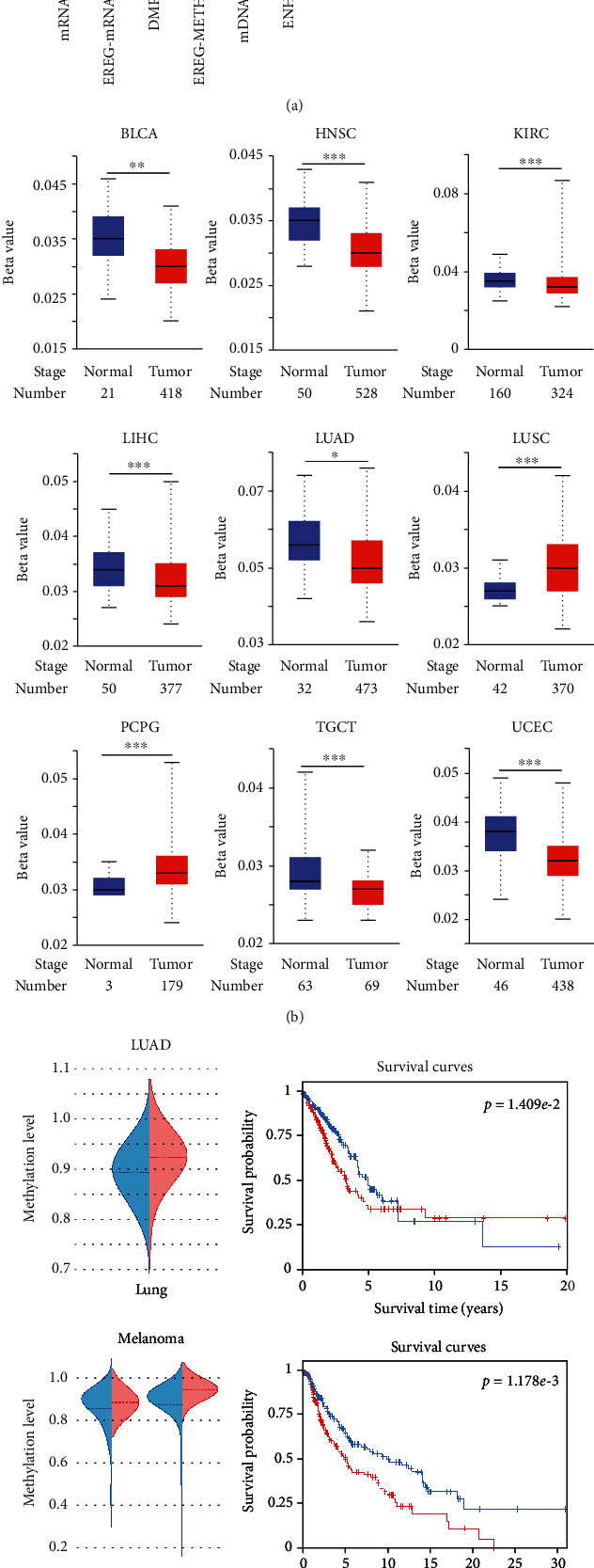
The relationship between KIF11 and cancer stemness, and epigenetic modification of KIF11 in pan-cancer. (a) The correlation between KIF11 expression and mRNAsi, EREG-mRNAsi, mDNAsi, DMPsi, ENHsi, and EREG-METHsi. ^∗^*P* < 0.05, ^∗∗^*P* < 0.01, ^∗∗∗^*P* < 0.001. (b) Boxplots show differential KIF11 promoter methylation level between tumors and paired normal tissues across TCGA database. (c) The methylation level of CpG island detected by probe cg05302035 between tumor and paired normal tissue (left panel) and the corresponding survival curves (right panel). The results were obtained from the EWAS database. (d) A forest plot shows the correlation between the methylation status of CpG island in KIF11 promoter and survival of patients with different cancer types.

Stemness, defined as the potential to self-renew and differentiate from a cell of origin, is a feature of precursor cells in the developing embryo [31]. As previous studies reported, gradual loss of the differentiated phenotype and gain of progenitor and stem-cell-like characteristics were the main reasons for driving tumor progression [32]. Analysis of the correlation between KIF11 expression and stemness indices, including mRNA expression-based stemness index (mRNAsi), epigenetically regulated-mRNA expression-based stemness index (EREG-mRNAsi), DNA methylation-based stemness index (mDNAsi), differentially methylated probes-based stemness index (DMPsi), enhancer elements/DNA methylation-based stemness index (ENHsi), and epigenetically regulated DNA methylation-based stemness index (EREG-METHsi), revealed that KIF11 expression correlated positively with cancer stemness in almost all human cancer types except THYM. KIF11 expression correlated positively with mRNAsi in 29 cancer types and negatively with mRNAsi in 2 cancer types, positively with EREG-mRNAsi in 19 cancer types and negatively with EREG-mRNAsi in 2 cancer types, positively with DMPsi in 15 cancer types and negatively with DMPsi in 2 cancer types, positively with EREG-METHsi in 14 cancer types and negatively with EREG-METHsi in 1 cancer type, positively with mDNAsi in 13 cancer types and negatively with mDNAsi in 2 cancer types, and positively with ENHsi in 10 cancer types and negatively with ENHsi in 2 cancer types (Figure 5(a)).

Epigenetic dysregulation of tumor cells frequently leads to oncogenic dedifferentiation and acquisition of stemness features [33, 34]. Compared with normal tissues, KIF11 promoter is hypomethylated in BLCA, HNSC, KIRC, LIHC, LUAD, TGCT, and UCEC and hypermethylated in LUSC and PCPG (Figure 5(b)). The results from the DiseaseMeth database further proved that KIF11 promoter is hypomethylated in bladder cancer, germ cell cancer, BLCA, ESCC, PAAD, HNSC, KICH, KIRC, LAML, LGG, OSC, OV, UCS, PA, PRAD, READ, and COAD and hypermethylated in gastric cancer, malignant pluripotent embryonal carcinoma, CHOL, ESCA, MM, GBM, LUSC, PCPG, and UCEC (Supplementary Figure 4). Furthermore, the methylation status of single CpG island and the correlation between methylation status and survival probability in different human cancers were analyzed using the EWAS database. The results indicated that the methylation status of single CpG island was associated with survival probability. Taking probe cg05302035 as an example, KIF11 was hypomethylated in LUAD, PTCC, and melanoma compared with normal tissue, and the hypomethylation status was related to survival probability (Figure 5(c)). The results from the MethSurv database indicated that the methylation status of single CpG island in KIF11 promoter was correlated with survival probability in different cancer types (Figure 5(d)). Details are shown in Supplementary Table 4. Collectively, these results indicated that KIF11 expression was strongly related to cancer stemness, and epigenetic methylation of KIF11 in patients was associated with prognosis across various human cancers.

### 3.5. KIF11 Correlated with Genomic Heterogeneity and Showed Characteristic Genetic Alteration in Pan-Cancer

**Figure 6:**
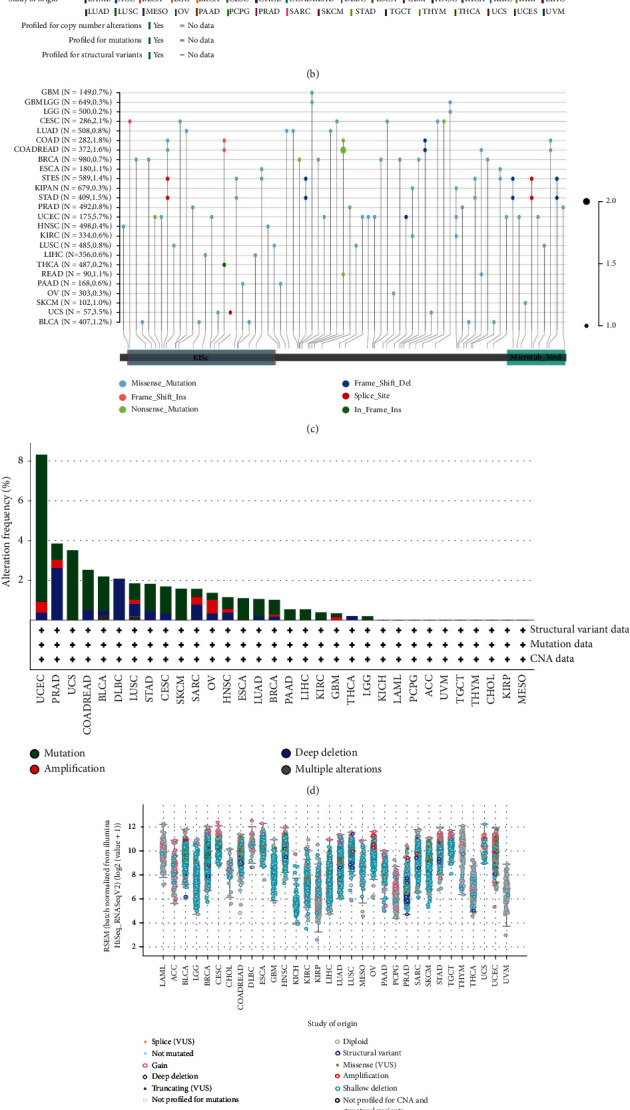
The relationship between KIF11 and genomic heterogeneity, and genetic alternation of KIF11 in pan-cancer. (a) The correlation between KIF11 expression and HRD, LOH, TMB, MSI, MATH, and ploidy. ^∗^*P* < 0.05, ^∗∗^*P* < 0.01, ^∗∗∗^*P* < 0.001. (b) Alteration landscape for KIF11 across multiple cancer types. (c) The number and distribution of different KIF11 mutations in various human cancer types. (d) KIF11 gene alteration frequency of different alteration types in cancer cohort. (e) KIF11 expression across different human cancer types with various gene alteration types. (f) The Kaplan-Meier curves of differences in overall survival, disease specific survival, and progression free survival between KIF11 altered group and KIF11 unaltered group.

Heterogeneity contributes to drug resistance and relapse after therapy, resulting in poor survival outcomes. Mutation profile differences among patients probably contribute to variability in response to chemotherapy and immunotherapy as first-line treatments [35]. KIF11 expression had significant associations with heterogeneity related factors, such as homologous recombination deficiency (HRD), loss of heterozygosity (LOH), tumor mutational burden (TMB), microsatellite instability (MSI), mutant allele tumor heterogeneity (MATH), and ploidy. KIF11 expression correlated positively with MATH in 8 cancer types and negatively with MATH in 5 cancer types, positively with ploidy in 9 cancer types and negatively with ploidy in 2 cancer types, positively with HRD in 20 cancer types and negatively with HRD in 3 cancer types, positively with LOH in 17 cancer types and negatively with LOH in 5 cancer types, positively with TMB in 19 cancer types and negatively with TMB in 2 cancer types, and positively with MSI in 10 cancer types and negatively with MSI in 5 cancer types (Figure 6(a)).

Genetic alterations induce changes in gene expression. We explored genetic alterations of KIF11 using cBioPortal, and the results indicated that genomic alteration of KIF11 occurred in 1.5% of patients across various cancer types. Among the different types of genetic alterations, amplification, deep deletion, truncating mutation, and missense mutation were the common types (Figure 6(b)). Detailed information about KIF11 mutations in different human cancer types indicated that the mutation sites of KIF11 distributed in the whole gene body including the KISc and microtubule binding domains. The highest alteration frequency of KIF11 was approximately 5.7% in patients with UCEC, and the lowest alteration frequency of KIF11 was approximately 0.2% in patients with THCA and LGG (Figure 6(c)). In addition, the results from the cBioPortal database indicated that UCEC patients had the highest KIF11 alteration frequency; THCA and LGG patients had lower KIF11 alteration frequency, and there is no KIF11 alteration in KICH, LAML, PCPG, ACC, UVM, TGCT, THYM, CHOL, KIRP, and MESO (Figure 6(d)). The types of KIF11 gene alterations were diverse, resulting in changes in gene expression (Figure 6(e)). The potential relationship between genetic alteration of KIF11 and the prognosis of patients indicated that tumor patients with genetic alterations in KIF11 showed better OS, DSS, and PFS than patients without alterations (Figure 6(f)). All these results indicated that KIF11 expression was strongly related to genomic heterogeneity and genetic alternation of KIF11 indeed occurred in many cancers and might play essential roles in cancer onset and progression.

### 3.6. Interaction Network and GO and KEGG Enrichment Analyses of KIF11

**Figure 7:**
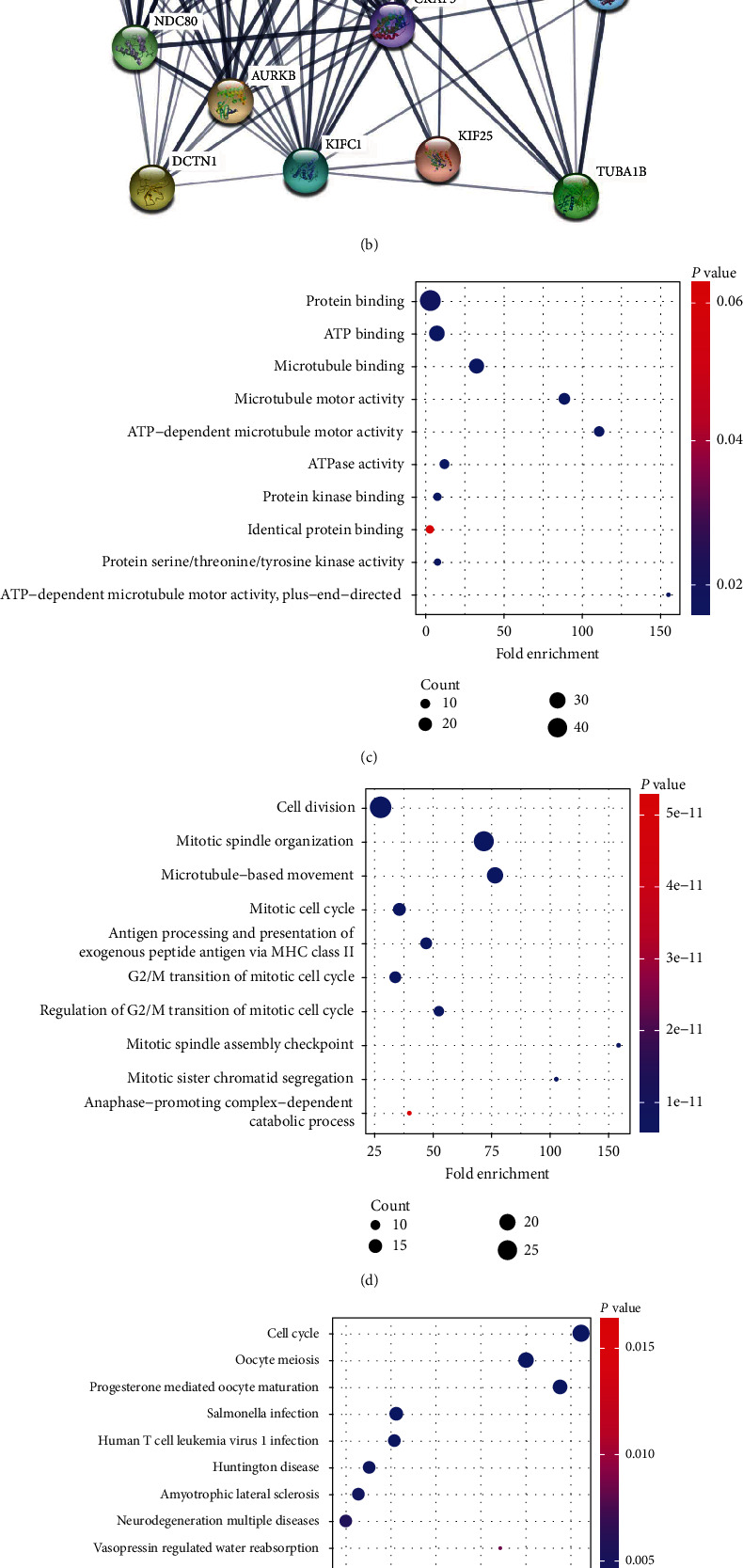
Interaction network and enrichment analysis of KIF11. (a) The gene-gene interaction network of KIF11 constructed using GeneMania database. (b) The protein-protein interaction network of KIF11 generated using STRING database. (c) GO analysis (molecular function) of 50 targeted binding proteins of KIF11. (d) GO analysis (biological process) of 50 targeted binding proteins of KIF11. (e) KEGG analysis of 50 targeted binding proteins of KIF11. (f) The correlation between KIF11 mRNA expression and cell cycle progression. The results were obtained from the HPA database using the single-cell RNA-sequencing data of the FUCCI U2OS cell line. (g) The correlation between KIF11 protein expression and cell cycle progression. The results were obtained from the HPA database using the data of indirect immunofluorescence assay of FUCCI U2OS cell line.

To better understand the function of KIF11 in cancer, we constructed the gene-gene interaction network for KIF11 by using GeneMania. Functional analysis suggested that the related genes were significantly associated with the cell cycle and antigen processing and presentation (Figure 7(a)). Next, a protein-protein interaction network of KIF11 was generated using the STRING database (Figure 7(b)). Fifty targeted binding proteins of KIF11 were screened out for GO and KEGG enrichment analyses. The result revealed that the molecular function was primarily involved in protein binding, ATP binding, microtubule binding, and microtubule motor activity (Figure 7(c)). The biological process was mainly enriched in cell division, cell cycle, antigen processing and presentation, and anaphase-promoting complex dependent catabolic process (Figure 7(d)). The KEGG pathway enrichment was mainly related to cell cycle, oocyte meiosis, pathogen infection, Huntington's disease, amyotrophic lateral sclerosis, neurodegeneration multiple diseases, vasopressin-regulated water reabsorption, and p53 signaling pathway (Figure 7(e)). Single-cell RNA-sequencing data from Fluorescent Ubiquitination-based Cell Cycle Indicator (FUCCI) U2OS cells revealed that KIF11 RNA expression was in relation to cell cycle progression (Figure 7(f)). Similarly, KIF11 protein expression level was correlated with interphase progression through the G1, S, and G2 phases (Figure 7(g)). The variation in protein and transcript expression of KIF11 consists with its role in cell cycle. Based on the above results, we hypothesized that KIF11 played essential roles in cell cycle, cell division, immune response, and p53 signaling pathway.

### 3.7. The Relationship between KIF11 and Molecular and Immune Subtypes

**Figure 8:**
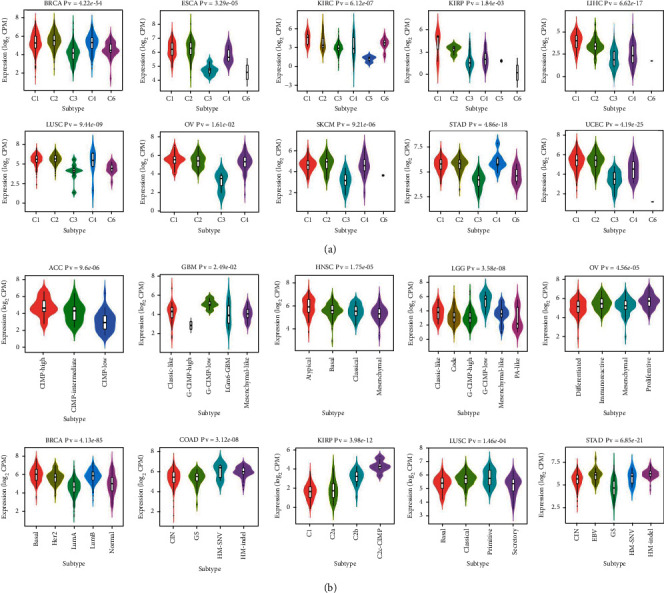
The relationship between KIF11 expression and immune/molecular subtypes in different human cancers. (a) The relationship between KIF11 expression and immune subtypes in BRCA, ESCA, KIRC, KIRP, LIHC, LUSC, OV, SKCM, STAD, and UCEC. (b) The correlation between KIF11 expression and molecular subtypes in ACC, GBM, HNSC, LGG, OV, BRCA, COAD, KIRP, LUSC, and STAD.

Previous studies have proved that tumor-infiltrating lymphocytes can affect patient survival. Thus, the role of KIF11 in immune and molecular subtypes among human cancers was investigated via the TISIDB database. And the results indicated that KIF11 expressed differently in different immune and molecular subtypes of various cancer types. For immune subtypes, KIF11 expressed at highest abundance in the immune subtype of C1 (wound healing) for KIRC, KIRP, LIHC, OV, UCEC, CHOL, GBM, KICH, LUAD, SARC, and UCS, C2 (IFN-gamma dominant) for BRCA, ESCA, LUSC, SKCM, STAD, BLCA, CESC, COAD, PAAD, PRAD, READ and TGCT, C3 (inflammatory) for HNSC, C4 (lymphocyte depleted) for ACC, LGG, MESO and UVM, C5 (immunologically quiet) for PCPG, and C6 (TGF-b dominant) for THCA (Figure 8(a) and Supplementary Figure 5A).

For molecular subtypes, ESCA, READ, and STAD expressed KIF11 at highest level in the molecular subtype of HM-INDEL, PRAD expressed KIF11 at highest level in the molecular subtype of 7-IDH1, HNSC expressed KIF11 at highest level in the molecular subtype of atypical, BRCA expressed KIF11 at highest level in the molecular subtype of basal, KIRP expressed KIF11 at highest level in the molecular subtype of C2c-CIMP, ACC expressed KIF11 at highest level in the molecular subtype of CIMP-HIGH, GBM and LGG expressed KIF11 at highest level in the molecular subtype of G-CIMP-LOW, COAD expressed KIF11 at highest level in the molecular subtype of HM-SNV, LIHC expressed KIF11 at highest level in the molecular subtype of iCluxter:1, PCPG expressed KIF11 at highest level in the molecular subtype of kinase signaling, SKCM expressed KIF11 at highest level in the molecular subtype of NF1-Any-Mutants, UCEC expressed KIF11 at highest level in the molecular subtype of pole, LUSC expressed KIF11 at highest level in the molecular subtype of primitive, and OV expressed KIF11 at highest level in the molecular subtype of proliferative (Figure 8(b) and Supplementary Figure 5B). These results suggested that KIF11 expression differed in molecular subtypes and immune subtypes of various human cancers.

### 3.8. The Relationship between KIF11 Expression and Neoantigens, ESTIMATE, and Immune Checkpoint Genes

**Figure 9:**
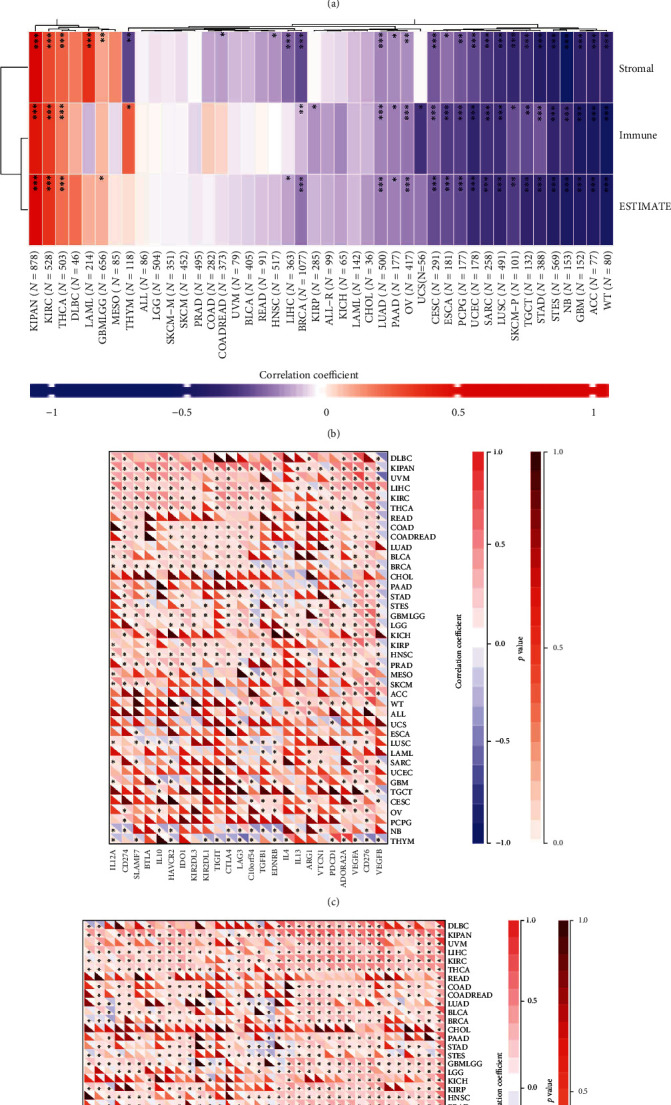
The relationship between KIF11 expression and neoantigen (a), ESTIMATE score (b), and immune checkpoint genes (c, d) in different human cancers. ESTIMATE: estimation of stromal and immune cells in malignant tumor tissues using expression data. ^∗^*P* < 0.05, ^∗∗^*P* < 0.01, ^∗∗∗^*P* < 0.001.

Tumor neoantigen is the repertoire of new unnatural proteins encoded by mutated genes of tumor that displays on the tumor cell surface, which could be specifically recognized by neoantigen-specific T cell receptors, and plays critical roles in T cell-mediated antitumor immune response and cancer immunotherapy [36]. The KIF11 expression was positively related to neoantigens in LUAD, BRCA, UCEC, SKCM, PRAD, LGG, and STAD (Figure 9(a)). To further explore the role of KIF11 in the immune response, the correlation between KIF11 expression and ESTIMATE was analyzed, and the results indicated that KIF11 expression correlated positively with ESTIMATE in KIPAN, KIRC, and THCA and negatively with ESTIMATE in ACC, WT, GBM, NB, STES, STAD, TGCT, SKCM-P, LUSC, SARC, UCEC, PCPG, ESCA, CESC, OV, PAAD, LUAD, and BRCA (Figure 9(b)). Overall, these results showed that KIF11 might participate in antitumor immunity by regulating the composition and immune mechanism in the TME.

Immune checkpoints are immune regulators of both stimulatory and inhibitory pathways and play an important role in immune cell infiltration and immunotherapy [37]. Subsequently, the association between KIF11 expression and immune checkpoint genes in human cancers was explored. Strong relationships between KIF11 expression and immune checkpoint genes were found in many human cancer types, including KIPAN, UVM, LIHC, KIRC, THCA, COAD, COADREAD, BLCA, BRCA, GBMLGG, LGG, KIRP, HNSC, PRAD, NB, and THYM. In KIPAN, UVM, LIHC, KIRC, THCA, COAD, COADREAD, BLCA, BRCA, GBMLGG, LGG, KIRP, HNSC, and PRAD, KIF11 expression was positively related to most immune checkpoint genes. In NB and THYM, KIF11 is negatively related with most immune checkpoint genes. For single immune checkpoint gene, the immune stimulator HMGB1 correlated positively with KIF11 in all human cancer types, and TNFSF4, BTN3A1, BTN3A2, and ENTPD1 correlated positively with KIF11 in most human cancer types. The immune inhibitor CD276 and VEGFA correlated positively with KIF11 in most human cancer types, but VEGFB correlated negatively with KIF11 in most human cancer types (Figures 9(c) and 9(d)). The above results suggested that KIF11 might coordinate the function of these immune checkpoint genes in different signal transduction pathways and potentially serve as an ideal pan-cancer biomarker for predicting the immunotherapy response.

### 3.9. The Correlation between KIF11 and Immune Cell Infiltration

**Figure 10:**
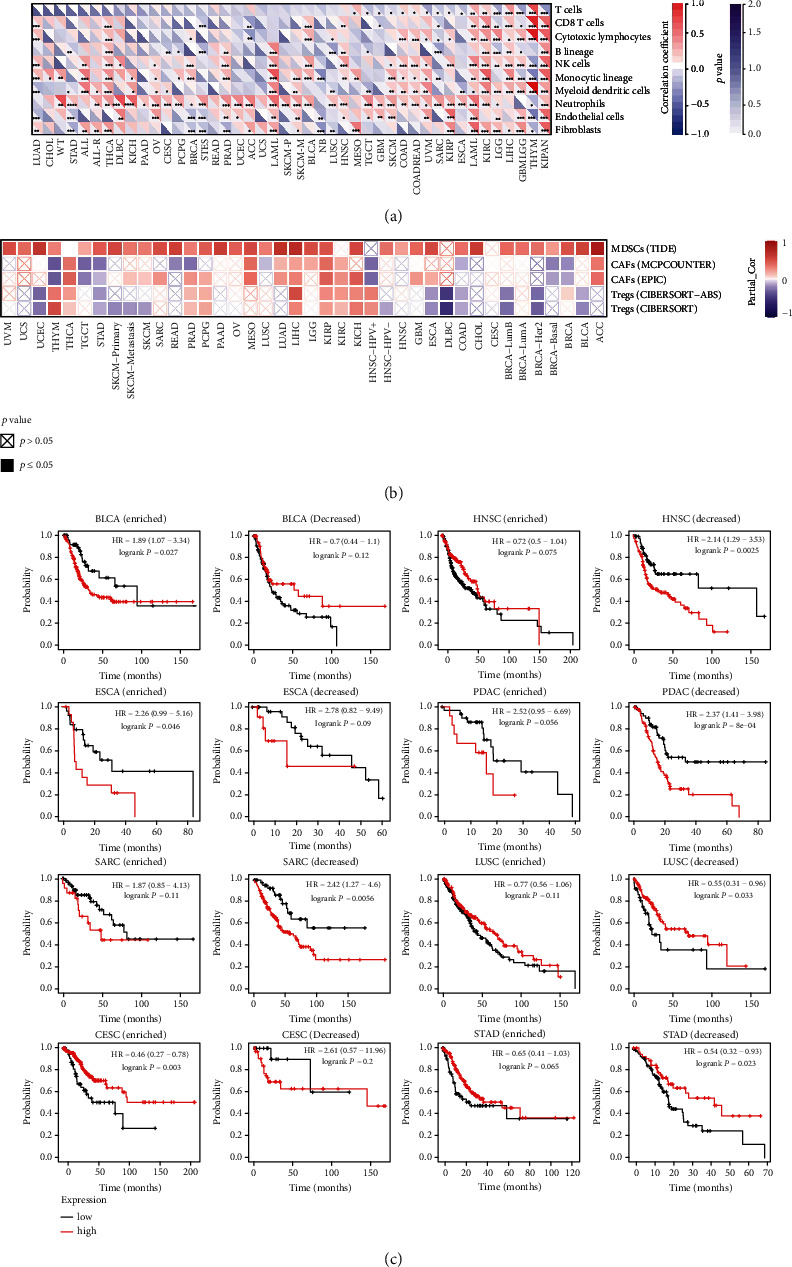
The relationship between KIF11 expression and immune infiltration. (a) The correlation between KIF11 expression and the infiltration of ten immune cell types in various human cancer types. ^∗^*P* < 0.05, ^∗∗^*P* < 0.01, ^∗∗∗^*P* < 0.001. (b) The correlation between KIF11 expression and the infiltration of three immunosuppressive cell types in various human cancer types. (c) The Kaplan-Meier plotter shows the correlation between KIF11 expression and OS in different CD4^+^ memory T cell subgroups in patients with different cancer types.

The above results indicated that KIF11 expressed differently in different immune subtypes, and KIF11 expression was strongly related to neoantigens, ESTIMATE, and immune checkpoint genes. Next, the relationship between KIF11 expression and immune cell infiltration was analyzed, and the result revealed that KIF11 expression had a strong relationship with T cell infiltration in 17 cancer types, CD8^+^ T cell infiltration in 15 cancer types, cytotoxic lymphocyte infiltration in 18 cancer types, B lineage cell infiltration in 16 cancer types, NK cell infiltration in 23 cancer types, monocytic lineage cell infiltration in 27 cancer types, myeloid dendritic cell infiltration in 24 cancer types, neutrophil infiltration in 35 cancer types, endothelial cell infiltration in 22 cancer types, and fibroblast infiltration in 22 cancer types (Figure 10(a)). The results from the TIMER database also proved that KIF11 expression had significant association with immune cell infiltration, and the detailed information was shown in Supplementary Table 5.

Furthermore, the correlation between KIF11 expression and infiltration of immunosuppressive cells that were known to promote T cell exclusion, such as MDSCs, CAFs, and Tregs, was analyzed using the TIMER database. KIF11 expression was positively correlated with tumor infiltration of MSDCs in most cancer types except CESC, DLBC, THCA, KIRC, and HPV positive HNSC, positively correlated with tumor infiltration of CAFs in ACC, KIRC, KIRP, LIHC, LUAD, MESO, and THCA and negatively correlated with tumor infiltration of CAFs in BRCA, HPV positive HNSC, STAD, TGCT, and THYM, and positively correlated with tumor infiltration of Tregs in HPV positive HNSC, KICH, KIRC, KIRP, LIHC, PCPG, PRAD, THCA, and THYM and negatively correlated with tumor infiltration of Tregs in BLCA, BRCA-Her2, BRCA-LumB, COAD, DLBC, ESCA, STAD, and UCEC (Figure 10(b)).

Since KIF11 expression was significantly correlated with immune infiltration, whether KIF11 expression affects the prognosis of patients because of immune infiltration was analyzed based on the expression level of KIF11 in various human cancers in related immune cell subgroups. The result indicated that KIF11 expression affected the prognosis of patients relying on various immune cell infiltrations. Taking CD4^+^ memory T cells as an example, BLCA and ESCA patients with high expression of KIF11 and enriched CD4^+^ memory T cells had a poor prognosis, while CESC patients with high expression of KIF11 and enriched CD4^+^ memory T cells had a better prognosis. SARC, HNSC, and PDAC patients with high expression of KIF11 and decreased CD4^+^ memory T cells had a poor prognosis, while LUSC and STAD patients with high expression of KIF11 and decreased CD4^+^ memory T cells had a better prognosis. On the contrary, there was no significant correlation between KIF11 expression and the prognosis of BLCA, ESCA, and CESC patients in the group with decreased CD4^+^ memory T cells and SARC, HNSC, PDAC, LUSC, and STAD patients in the group with enriched CD4^+^ memory T cells (Figure 10(c)). The detailed information was shown in Supplementary Table 6. These results indicated that KIF11 might affect the prognosis of patients in part due to immune infiltration.

### 3.10. The Association between KIF11 and Therapeutic Response in Multiple Cancer Types

**Figure 11:**
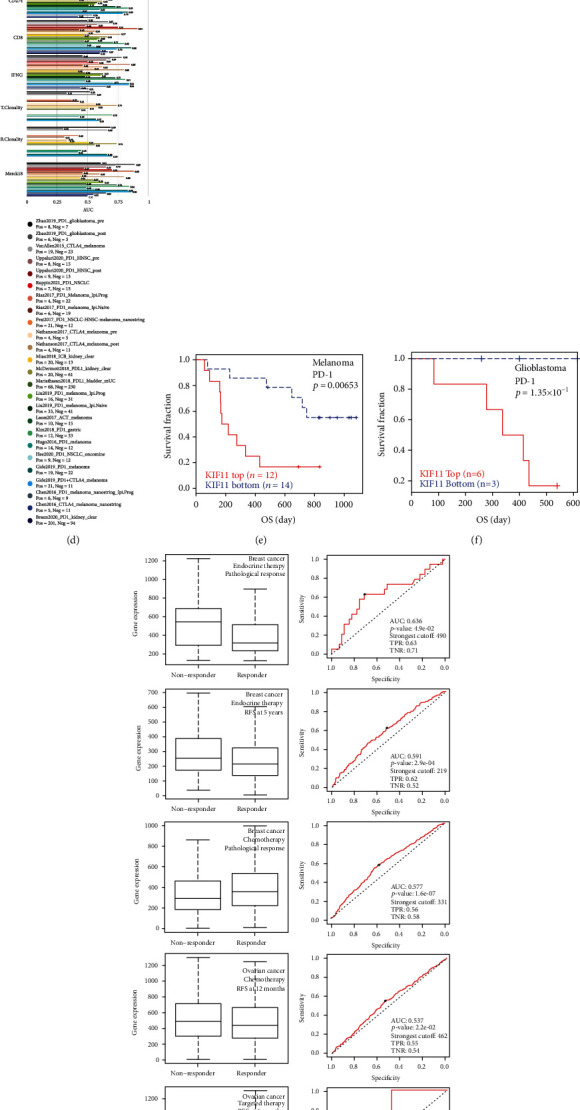
The relationship between KIF11 expression and therapeutic response in multiple cancer types. (a–c) The correlation between drug sensitivity and the CNV (a), methylation (b), expression (c) of KIF11 analyzed by the RNAactDrug database. (d) The biomarker relevance of KIF11 compared to standardized biomarkers in immune checkpoint blockade (ICB) subcohorts. (e, f) The Kaplan-Meier curves as a measure of the PD-1 ICB therapy response between cancer cohorts with high and those with low expression levels of KIF11, melanoma (e), and glioblastoma (f). (g) The receiver operating characteristic (ROC) curve of the correlation between KIF11 expression and response to endocrine therapy in breast cancer cohorts, chemotherapy in breast and ovarian cancer cohorts, targeted therapy in ovarian cancer cohorts.

Considering the role of KIF11 in tumor progression and immune cell infiltration, we verified the relationship between KIF11 expression and therapeutic response in different tumors. The results from the RNAactDrug database showed that the expression, methylation, and CNV of KIF11 were strongly related to drug sensitivity (Figures 11(a)–11(c) and Supplementary Table 7). The biomarker relevance of KIF11 was compared with standardized biomarkers based on their predictive power on overall survival and response outcome of immune checkpoint blockade (ICB) subcohorts. KIF11 had an area under the receiver operating characteristic curve (AUC) of >0.5 in 12 of the 23 ICB subcohorts. KIF11 had a higher predictive value than T. Clonality, B. Clonality, and TMB, which had an area under the receiver operating characteristic curve (AUC) of >0.5 in 9, 7, and 8 of the 23 ICB subcohorts, respectively but lower than MSI score (AUC > 0.5 in 13 ICB subcohorts), CD8 (AUC > 0.5 in 18 ICB subcohorts), IFNG (AUC > 0.5 in 17 ICB subcohorts), CD274 (AUC > 0.5 in 21 ICB subcohorts), Merck18 (AUC > 0.5 in 18 ICB subcohorts), and TIDE (AUC > 0.5 in 18 ICB subcohorts) (Figure 11(d)). Furthermore, lower expression level of KIF11 was associated with clinical benefits of programmed death receptor-1 (PD-1) ICB therapy in patients with melanoma and glioblastoma (Figures 11(e) and 11(f)). In addition, KIF11 expression was correlated with therapeutic response in clinical cancer cohorts. Breast cancer patients with higher KIF11 expression were resistant to endocrine therapy and were resistant to chemotherapy with lower KIF11 expression. Ovarian cancer patients with higher KIF11 expression were resistant to chemotherapy and targeted therapy (Figure 11(g)). Taken together, those results indicated that KIF11 might serve as an ideal biomarker for predicting the therapeutic response.

### 3.11. KIF11 Gene Coexpression Network

**Figure 12:**
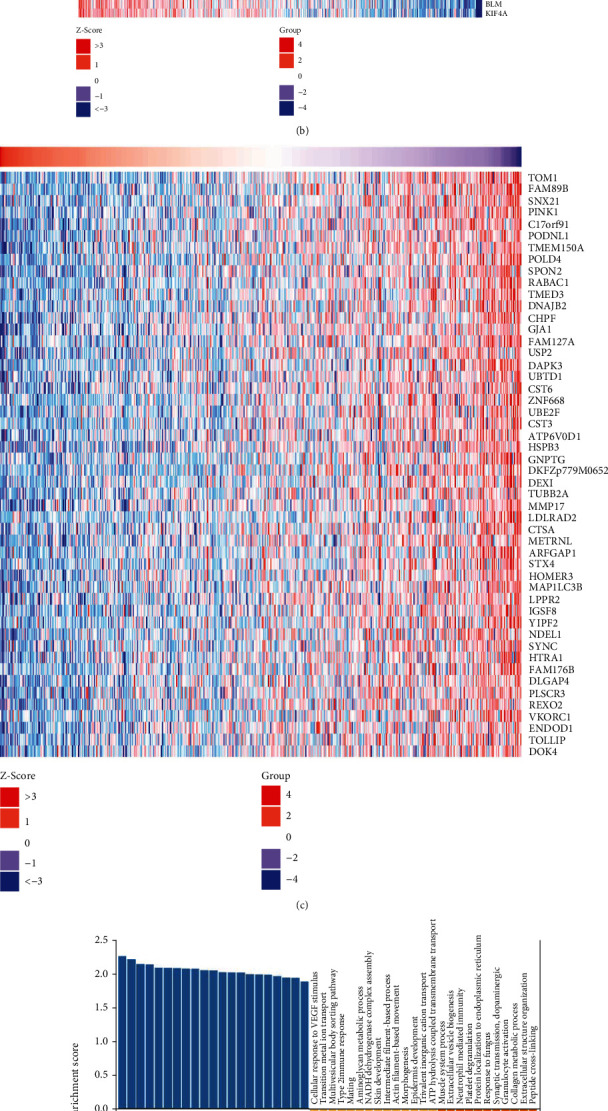
The KIF11 coexpression genes in HNSC. (a) Highly correlated genes of KIF11 in HNSC cohort tested by Spearman's correlation. (b) Heat map shows the top 50 genes positively correlated with KIF11 in HNSC. (c) Heat map shows the top 50 genes negatively correlated with KIF11 in HNSC cohort. (d) Bar chart of KIF11 GO analysis (biological process) in HNSC cohort. (e) Volcano plot of KIF11 KEGG pathways in HNSC cohort.

The above results indicated that KIF11 was strongly correlated with the prognosis, immunity, and therapeutic response. Next, KIF11 coexpression network in HNSC was identified using the LinkedOmics database to verify the molecular mechanisms affected by KIF11. In HNSC, 5975 genes (red dots) were positively related to KIF11, and 5568 genes (green dots) were negatively related to KIF11 (*P* value < 0.05) (Figure 12(a)). Heat maps displayed the top 50 genes positively and negatively correlated with KIF11 (Figures 12(b) and 12(c)). The detailed information was shown in Supplementary Table 8. KIF20B, MKI67, and ASPM had the strongest association with KIF11 (*r* = 0.84, 0.82, 0.80, and *P* = 8.11*E* − 139, 3.85*E* − 125, 7.23*E* − 116, respectively). Furthermore, gene set enrichment analysis (GSEA) was used to determine the main GO terms of KIF11 coexpression genes. KIF11 and the coexpression genes primarily participated in DNA replication, chromosome segregation, DNA repair, DNA recombination, cell division, cell cycle, and chromosome organization (Figure 12(d)). Similarly, the KEGG pathway analysis showed that KIF11 and the coexpression genes were enriched in cell cycle, DNA replication, homologous recombination, DNA repair, cell division, p53 signaling pathway, platinum drug resistance, and microRNAs in cancer (Figure 12(e)). These data furtherly demonstrated that KIF11 might play an essential role in human cancers by regulating cell division, cell cycle, chromosome organization, DNA repair, p53 signaling pathway, and drug resistance.

## 4. Discussion

KIF11 is a motor protein that plays critical roles in bipolar spindle establishing during mitosis. Consisting with its role in cell division, numerous studies showed that KIF11 was implicated in human tumor [38]. However, the oncological role of KIF11 has not been comprehensively explored in pan-cancer. In this study, we comprehensively interrogated the role of KIF11 in human cancers.

In the first step of our study, we carefully analyzed the expression level of KIF11 in different tumors and normal tissues using the TIMER database, GEPIA2 database, UALCAN database, HPA database, SangerBox website, and GeneCards database. The results indicated that KIF11 expression was significantly higher in most tumors compared with normal tissues. The immunohistochemical staining results furtherly confirmed the above results. Comparing the expression level of KIF11 across different normal tissues showed that KIF11 expressed at low level in most tissues but high in immune related tissues, such as thymus, bone marrow, tonsil, and lymph node. On the contrary, KIF11 expressed at high level in cancer cell lines generally. The immunohistochemical staining results furtherly confirmed that lymph node showed higher KIF11 expression level in both normal and tumor tissues. Previous studies showed that KIF11 played a precise role in lymphatic vascular development and function. Heterozygous mutations in KIF11 lead to abnormal lymphedema in microcephaly, lymphedema, and chorioretinal dysplasia (MLCRD) [39]. An important aspect of lymphedema is the disruption to lymphatic fluid transport function and immune cell trafficking that ultimately results in impaired immunity [39, 40]. Those results suggested that KIF11 indeed promoted oncogenesis and tumor progression. Furthermore, the function of KIF11 in lymph system implied the possibility that KIF11 might influence tumor progression through immunity indirectly.

Next, the relevance between KIF11 expression and prognosis was analyzed. High KIF11 expression was associated with poorer prognosis in most human cancer types except THYM. Similarly, KIF11 expression was previously reported to be associated with shorter survival time in patients with LUAD and PAAD [41, 42]. Those results indicated that KIF11 was a potential pan-cancer prognostic biomarker. Stemness was defined as the potential to self-renew and differentiate from the cell of origin [31]. Subpopulations of cancer cells, which were termed as cancer stem cells or stem-like cancer cells, have been isolated from various cancer patients and found to have high stemness properties [43, 44]. Previous studies reported that gradual loss of the differentiated phenotype and gain of progenitor and stem-cell-like characteristics were the main reasons for driving tumor progression [31, 32]. Our results indicated that KIF11 expression correlated positively with cancer stemness in almost all human cancer types except THYM. KIF11 expression correlated negatively with DMPsi, EREG-METHsi, mDNAsi, and ENHsi in THYM. This result might provide a possible reason for the above puzzle that why THYM patients with high KIF11expression showed an opposed survival probability compared with other cancer patients with high KIF11expression. Epigenetic dysregulation of tumor cells frequently leads to oncogenic dedifferentiation and acquisition of stemness features [33, 34]. DNA methylation is a major form of epigenetic modification that generally suppresses the gene expression [45]. Our results indicated that KIF11 promoter was hypomethylated in BLCA, HNSC, KIRC, LIHC, LUAD, UCEC, PAAD, LGG, OV, UCS, PRAD, READ, and COAD, which was consistent with the upregulation of the KIF11 expression. Undifferentiated primary tumors are more likely to result in the spread of cancer cells to distant organs, causing disease progression and poor prognosis [34, 46, 47]. As we can see in Figure 3, a roughly gradient increase of KIF11 expression along with the progression of pathological stages and nodal metastasis was observed in many patients with different tumors. Those results suggested a strong relationship between epigenetic modification, gene expression, cancer stemness, disease progression, and prognosis.

Cancer is a multistage process that has been characterized by a series of chromosomal changes. Some cancers accumulated many chromosomal rearrangements and most likely an even greater number of changes in the tumor DNA sequence [48]. Our results indicated that genomic alteration of KIF11 occurred in 1.5% of patients with various cancer types. The mutation points of KIF11 distributed in the whole gene body including the KISc and microtubule binding domains. The results analyzed by the SangerBox website and cBioPortal database indicated that UCEC patients had the highest KIF11 alteration frequency; THCA and LGG patients had lower KIF11 alteration frequency, and there is no KIF11 alteration in KICH, LANL, PCPG, ACC, UVM, TGCT, THYM, CHOL, KIRP, and MESO. Cancer is a highly heterogeneous disease with unique phenotypic and genomic features that differ among individual patients and even among individual tumor regions [49]. Heterogeneity resulting from clonal expansion of variability of gene expression, genomic alteration, and individual mutation among tumors forms the basis of the complexity of cancer [35]. We analyzed the relationship between KIF11 expression and genomic heterogeneity. The results indicated that KIF11 expression correlated positively with genomic heterogeneity in ACC, STES, STAD, LUSC, GBM, SARC, LUAD, BRCA, BLCA, PAAD, LIHC, UVM, MESO, KICH, LGG, and KIRC but negatively with genomic heterogeneity in THYM. The results consisted with the relationship between KIF11 expression and prognosis in THYM, which could bring us a hint to understand the function of KIF11 in THYM.

Tumors comprise a complex, diverse, and integrated ecosystem of relatively differentiated cancer cells, stem-like cancer cells, infiltrating immune cells, endothelial cells, cancer-associated fibroblastic cells, endothelial cells, pericytes, and so on [3, 34]. The multifaceted functions of the noncancerous cells in the tumor regulate the growth of cancer cells. In addition, the proteins or metabolites that presented by these cells may influence the tumor progression. The cancer microenvironment may serve as the ecology in which cancer cells were selected for proliferation and survival [3, 50]. Our study found that KIF11 correlated negatively with immune, stromal, and ESTIMATE scores of the TME in most human cancer types but correlated positively with immune, stromal, and ESTIMATE scores of the TME in KIPAN, KIRC, and THCA. These results indicated that KIF11 played a different regulatory role in tumor purity across various tumors. The tumor microenvironment provides numerous opportunities for cell-cell signals to modulate tumor progression [34, 51]. It is necessary to understand the regulatory mechanism of the interaction between heterogeneous cancer cells within the cancer population as well as their interaction with the noncancerous cells present within or adjacent to the cancer cells [3]. Neoantigen and immune checkpoint molecular are main regulators of the interaction between different cells in tumor. Our results showed that KIF11 correlated positively with neoantigen in LUAD, BRCA, UCEC, STAD, SKCM, PRAD, LGG, and immune checkpoint genes in most human tumors but negatively with most immune checkpoint genes in NB and THYM. The above results also proved that KIF11 was closely related with the TME in human tumors and played different regulation roles in various tumors.

The composition and abundance of tumor-infiltrating immune cells in the tumor microenvironment have been proved to be an independent predictor of cancer patient prognosis, immunotherapeutic response and efficacy [52]. The infiltration of tumors and their metastases by immune cells can contribute both positively and negatively to disease progression and clinical outcomes [53]. These different outcomes are correlated with the diversity of lymphocytes infiltrating neoplastic lesions [54]. Our study demonstrated that KIF11 had a strong association with immune cell infiltration in the TME. KIF11 showed a positive relationship with neutrophil infiltration in most human tumors. Previous studies have reported that neutrophils make up a substantial proportion of the immune infiltrate in a wide variety of cancer types and are active players in the immune response to malignancy [55]. The role of neutrophil in tumors is intricate. Some studies suggested that tumor-associated neutrophil had various antitumor functions, such as direct cytotoxicity towards cancer cells and inhibition of metastasis [56, 57]. Conversely, numerous other studies indicated that tumor-associated neutrophils were capable of supporting tumor progression through stimulating tumor cell invasion, migration and motility, promoting the angiogenic switch, and modulating other immune cells [58, 59]. In the past few years, researchers have recognized that cancer-related neutrophil is able to retain functional plasticity and can undergo functional remodeling when exposed to various cues in the TME [60]. KIF11 may play multifaceted roles in tumor by influencing the infiltration of neutrophil. Recent studies have demonstrated that MDSCs also infiltrate tumors, inhibit dendritic cell and T cell function and number, and facilitate tumor growth, metastasis, and angiogenesis [54]. In our study, we found that KIF11 correlated positively with MDSCs in most human tumors. This result indicated that KIF11 might support tumor progression by promoting the infiltration of MDSCs. Since KIF11 expression was significantly correlated with immune infiltration, we next explored whether KIF11 expression affected the prognosis of patients because of immune infiltration based on the expression level of KIF11 in various human cancers in related immune cell subgroups. The result indicated that KIF11 indeed affected the prognosis of patients relying on various immune cell infiltrations.

Because of the important role in mitosis, KIF11 has been a target for development of potential anticancer drugs [61]. Our results indicated that KIF11 showed strong relationship with drug sensitivity. For example, KIF11 expression correlated positively with drug sensitivity for trametinib, refametinib, and tanespimycin but negatively with drug sensitivity for navitoclax, topotecan, and vorinostat. KIF11 methylation correlated positively with drug sensitivity for selumetinib, afatinib, and trametinib but negatively with drug sensitivity for axitinib, talazoparib, and olaparib. KIF11 CNV correlated positively with drug sensitivity for tivozanib, masitinib, and quizartinib but negatively with drug sensitivity for trametinib. The above results indicated that KIF11 was a valuable reference index for clinical anticancer drug selection. Cancer immunotherapy has changed the treatment landscape for cancer patients. Immune checkpoint inhibitors that block the immunosuppressive receptors such as cytotoxic T-lymphocyte-associated antigen 4 (CTLA-4) and PD-1 can reverse the dampened antitumor immune response of T cells in the tumor microenvironment and trigger anticancer properties of infiltrating T cells [62]. Our results showed that KIF11 was a powerful cancer immune evasion biomarker compared with standardized biomarkers in immune checkpoint blockade subcohorts. Furthermore, lower expression level of KIF11 was associated with clinical benefits of PD-1 ICB therapy in melanoma and glioblastoma. In addition, KIF11 expression was correlated with therapeutic response in clinical cancer cohorts. Those results together indicated that KIF11 might serve as an ideal biomarker for predicting the therapeutic response and outcome.

In our analysis of KIF11 interaction and coexpression networks, we found that KIF11 and its partners mainly took part in regulating the cell division, cell cycle, p53 signaling pathway, microRNAs in cancer, platinum drug resistance, DNA repair and recombination, chromatin organization, and antigen processing and presentation via MHC class II. Consisting with previous studies, KIF11's partners furtherly confirmed that KIF11 was responsible for cell division. KIF11 expression was related to cell cycle progression. In addition, some of KIF11's partners play roles in cell cycle. Those results indicated that KIF11 might also take part in interphase progression through the G1, S, and G2 phases. There is no doubt that human cancers display many mutations, and the genetic alternation can be obtained by factors internal to cancer cells, including DNA repair deficiencies, abnormal DNA recombination, and deficiencies in chromatin organization. Our results showed that KIF11 expression was correlated with genomic heterogeneity. The interaction and coexpression networks of KIF11 furtherly confirmed that KIF11 was an important influencing factor of genomic heterogeneity in human tumors. The p53 signaling pathway is a classic cancer-related signaling pathway. KIF11 expression showed a positive relationship with p53 mutation. In addition, KIF11 partners were also documented to be involved in p53 signaling pathway. Those results together confirmed that KIF11 might also influence tumor initiation, development, and progression by p53 signaling pathway.

Immune checkpoint inhibition and other types of immunotherapy have led to impressive gains in survival for many tumor patients. Immune checkpoint inhibition efficacy requires tumor antigens to be recognized by tumor-infiltrating T cells which are mediated by T cell receptor and MHC interaction. MHC-II molecules are primarily expressed in professional antigen presenting cells such as macrophages, dendritic cells, and B cells and predominantly present exogenously-derived peptide antigens to CD4^+^ T cells [63–65]. MHC-II and related pathway components have been found to be expressed by cancer cells in various human tumors including glioma, prostate cancer, breast cancer, ovarian cancer, classic Hodgkin's lymphoma, colorectal cancer, melanoma, and non-small cell lung cancer [65]. The MHC-II expression in tumors has been associated with increased formation of tertiary lymphoid structures, higher number of both CD4^+^ and CD8^+^ tumor-infiltrating lymphocytes, upregulation of genes associated with IFN*γ* pathway activation, absence of lymphovascular invasion, higher levels of *IFNG*, *IL2*, and *IL12* mRNA, and improved survival including response to immune checkpoint inhibition, increased tumor-infiltrating lymphocytes, and proinflammatory IFN signaling in human tumors [63, 65]. Our results confirmed that KIF11 and its partners might play roles in antigen processing and presentation via MHC-II, which suggested that KIF11 indeed closely related with immune cell infiltration in human tumors.

## 5. Conclusions

In the present study, we conducted a comprehensive analysis of the KIF11 gene based on multiomics data and investigated the roles of KIF11 in oncogenesis, progression, tumor immune infiltration, and therapy outcome from the perspective of pan-cancer. In conclusion, our study evaluated the prognostic and immunological value of KIF11 in pan-cancer. KIF11 expression was significantly upregulated in tumors and showed strong relationships with pathological stage and prognosis across different cancer types. The expression of KIF11 was diverse in different immune subtypes and remarkably correlated with ESTIMATE, immune checkpoint, and immune cell infiltration in the tumor microenvironment. Meanwhile, KIF11 was associated with drug sensitivity and could serve as a powerful biomarker for predicting immune, targeted, and chemotherapeutic outcomes in different cancers. Taken together, our study revealed that KIF11 might serve as a potential pan-cancer biomarker for cancer detection, prognosis, therapy design, and follow up. However, we also noticed that the results lack validation of clinical specimens and biological experiments, which is the limitation of this study. Further experiments in vivo and in vitro should be performed in future studies that may present a more convincing viewpoint according to the results.

## Data Availability

All data generated or analyzed during this study are included in the manuscript and supporting files.

## Supplementary Materials

Supplementary 1 Supplementary Figure 1: the expression level of KIF11 in tumors and normal tissues. (A) KIF11 expression level in different cancer types and normal tissues analyzed by the UALCAN online tool. ^∗^*P* < 0.05, ^∗∗^*P* < 0.01, ^∗∗∗^*P* < 0.001. (B) KIF11 expression level in different normal tissues analyzed by the SangerBox website. (C) KIF11 expression level in different cancer cell lines analyzed by the SangerBox website. (D) KIF11 protein level in different normal tissues analyzed by the GeneCards database. (E) KIF11 protein level in different cell lines analyzed by the GeneCards database.

Supplementary 2 Supplementary Figure 2: the expression level of KIF11 in different TP53 mutation status.

Supplementary 3 Supplementary Figure 3: the Kaplan-Meier survival curve of human cancers with high and low KIF11 expression analyzed by the Kaplan-Meier plotter database.

Supplementary 4 Supplementary Figure 4: boxplots show differential KIF11 promoter methylation level between tumors and paired normal tissues analyzed by the DiseaseMeth database.

Supplementary 5 Supplementary Figure 5: the relationship between KIF11 expression and immune/molecular subtypes in different human cancers.

Supplementary 6 Supplementary Table 1: abbreviations.

Supplementary 7 Supplementary Table 2: cancer tissue material and clinicopathological data.

Supplementary 8 Supplementary Table 3: the association between KIF11 expression and survival of patients with different cancer types analyzed by the PrognoScan database.

Supplementary 9 Supplementary Table 4: the relationship between the methylation status of single CpG island of KIF11 and survival of patients with different cancer types.

Supplementary 10 Supplementary Table 5: the relationship between KIF11 expression and pan-cancer immune cell infiltration analyzed by the TIMER database.

Supplementary 11 Supplementary Table 6: prognostic value of KIF11 expression according to different immune cell subgroups.

Supplementary 12 Supplementary Table 7: the relationship between drug sensitivity and the expression, methylation, and CNV of KIF11 analyzed by the RNAactDrug database.

Supplementary 13 Supplementary Table 8: the KIF11 coexpression genes in HNSC cohort.
