# Supplementary figures and images for: KIF11 As a Potential Pan-Cancer Immunological Biomarker Encompassing the Disease Staging, Prognoses, Tumor Microenvironment, and Therapeutic Responses
Source: Oxid Med Cell Longev. 2022 Dec 16;2022:2764940. doi: 10.1155/2022/2764940 (PMC9893523; doi:10.1155/2022/2764940)

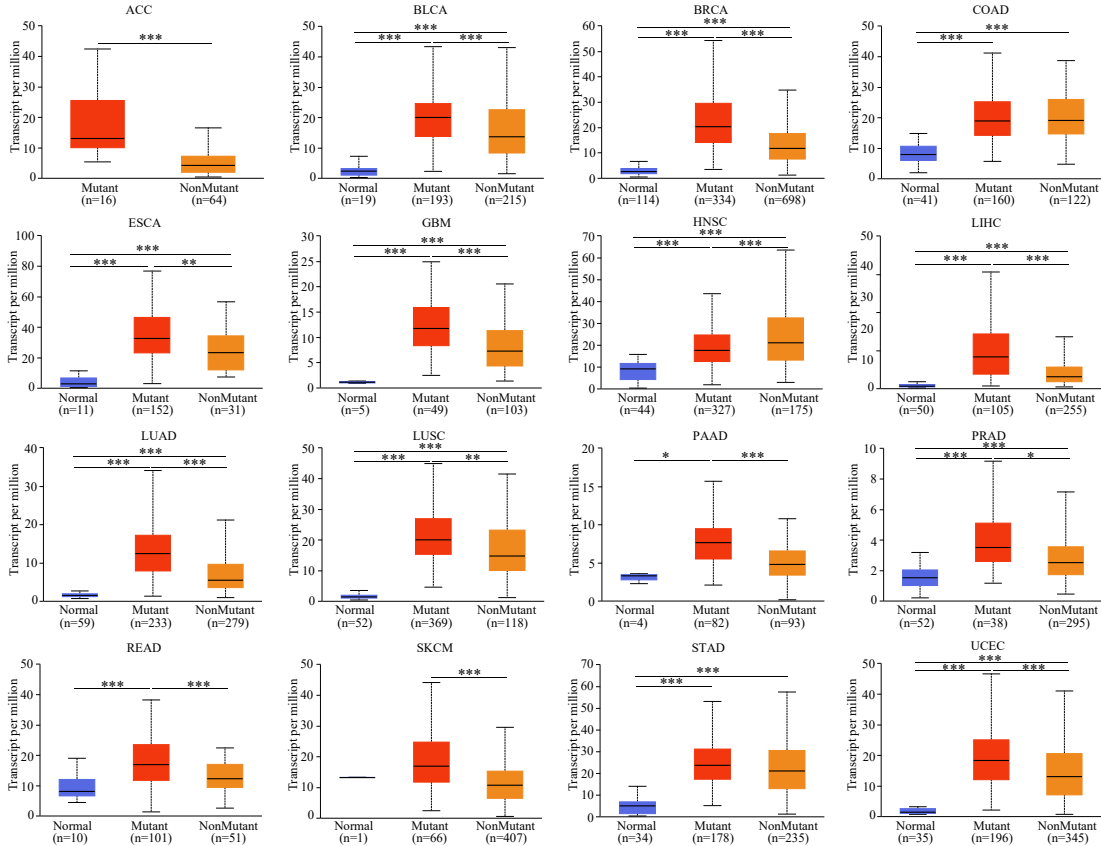

Supplement: Supplementary 2 — Supplementary Figure 2: the expression level of KIF11 in different TP53 mutation status. [file 2764940.f2.pdf]

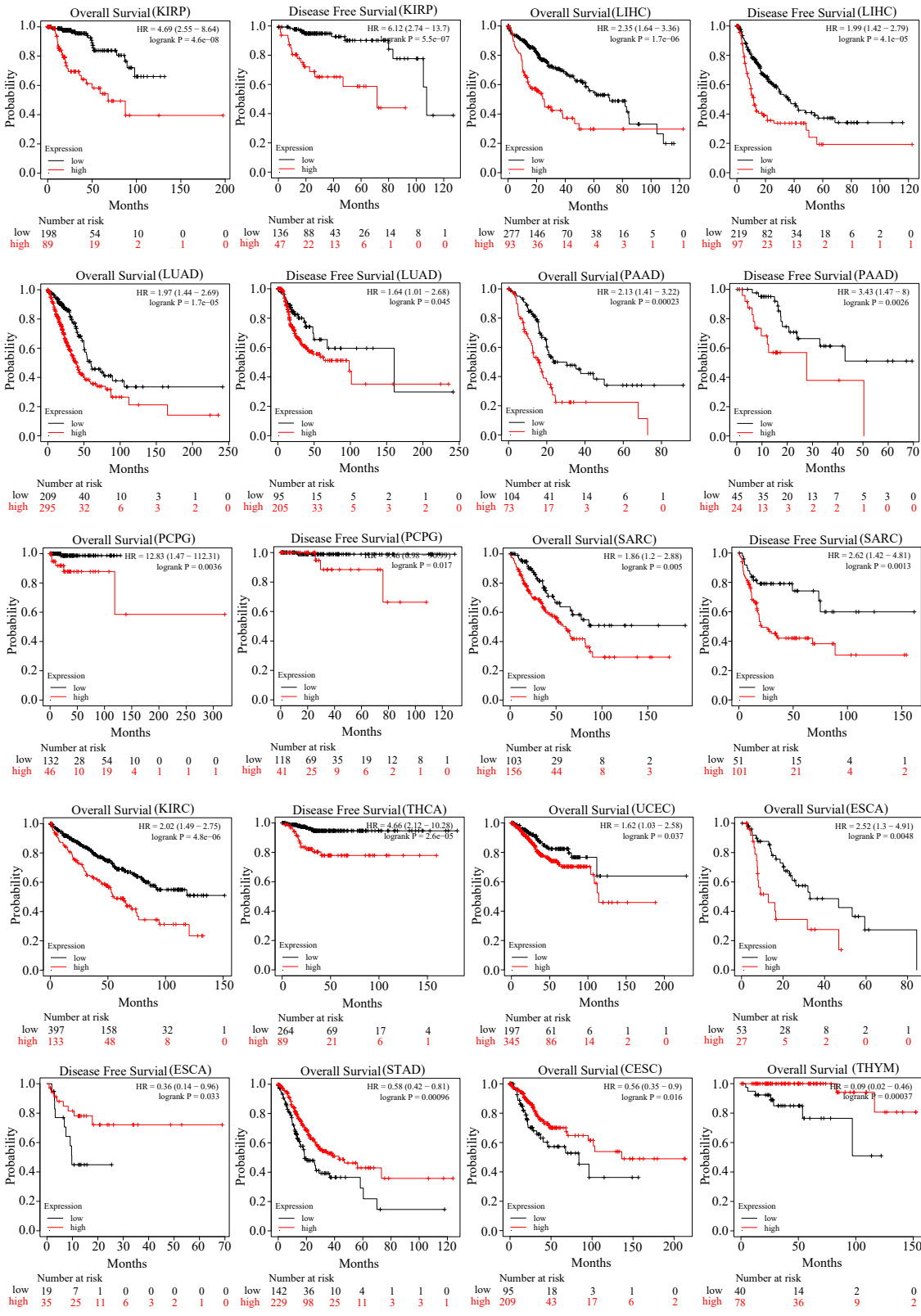

Supplement: Supplementary 3 — Supplementary Figure 3: the Kaplan-Meier survival curve of human cancers with high and low KIF11 expression analyzed by the Kaplan-Meier plotter database. [file 2764940.f3.pdf]

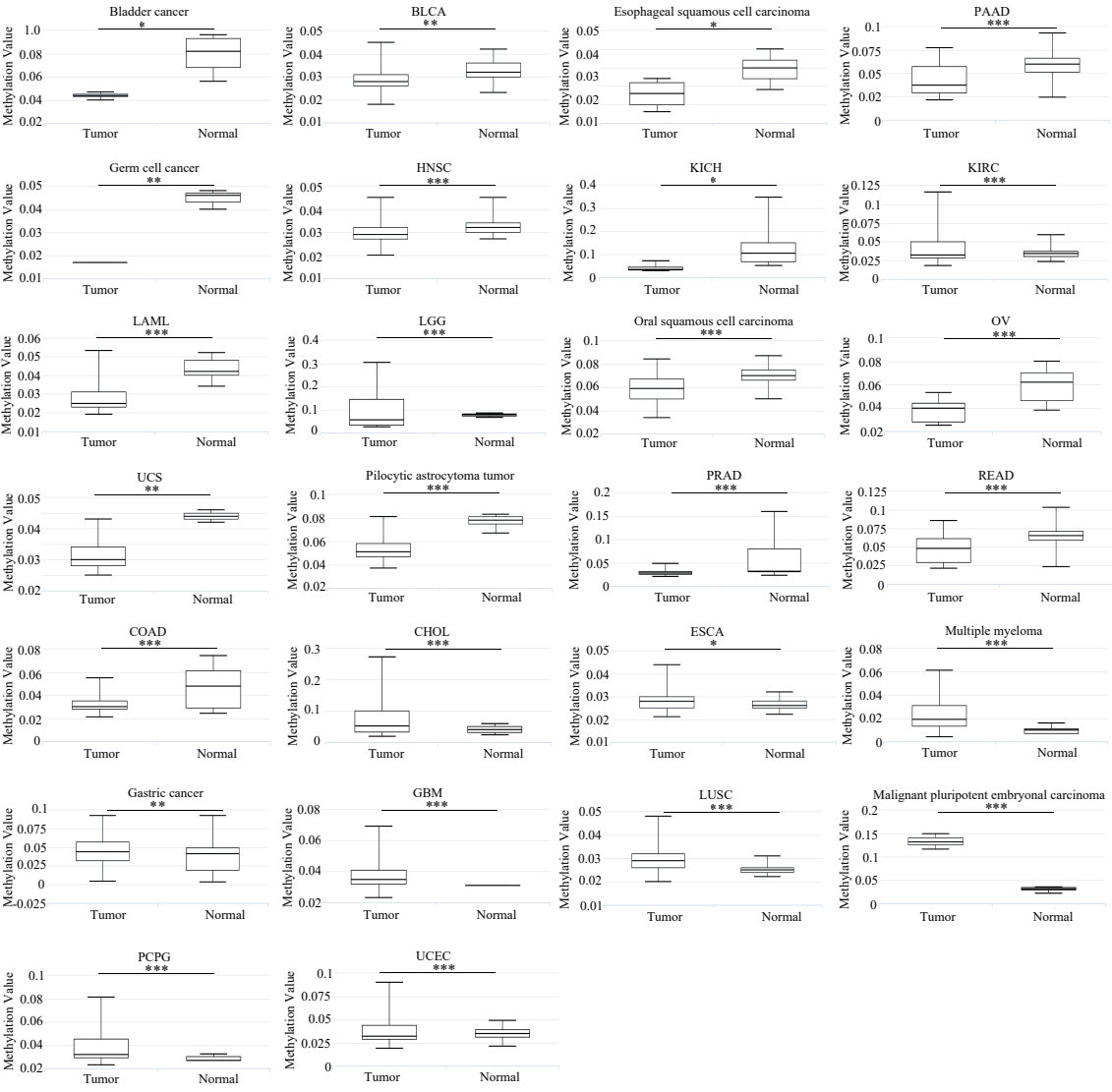

Supplement: Supplementary 4 — Supplementary Figure 4: boxplots show differential KIF11 promoter methylation level between tumors and paired normal tissues analyzed by the DiseaseMeth database. [file 2764940.f4.pdf]

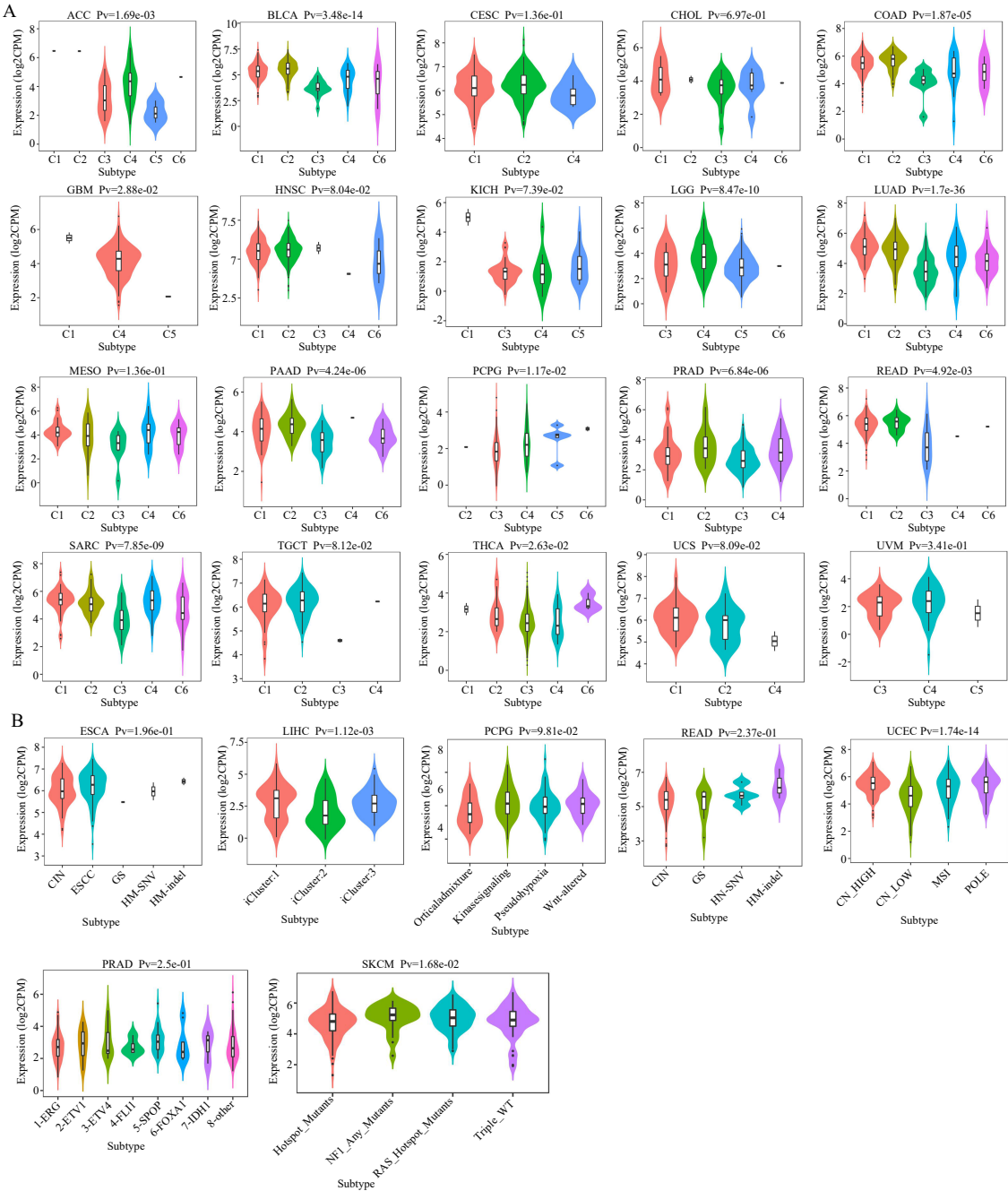

Supplement: Supplementary 5 — Supplementary Figure 5: the relationship between KIF11 expression and immune/molecular subtypes in different human cancers. [file 2764940.f5.pdf]
