# Supplementary material for: KIF11 As a Potential Pan-Cancer Immunological Biomarker Encompassing the Disease Staging, Prognoses, Tumor Microenvironment, and Therapeutic Responses
Source: Oxid Med Cell Longev. 2022 Dec 16;2022:2764940. doi: 10.1155/2022/2764940 (PMC9893523; doi:10.1155/2022/2764940)
Supplement: Supplementary 1 — Supplementary Figure 1: the expression level of KIF11 in tumors and normal tissues. (A) KIF11 expression level in different cancer types and normal tissues analyzed by the UALCAN online tool. ∗P < 0.05, ∗∗P < 0.01, ∗∗∗P < 0.001. (B) KIF11 expression level in different normal tissues analyzed by the SangerBox website. (C) KIF11 expression level in different cancer cell lines analyzed by the SangerBox website. (D) KIF11 protein level in different normal tissues analyzed by the GeneCards database. (E) KIF11 protein level in different cell lines analyzed by the GeneCards database. [file 2764940.f1.pdf]

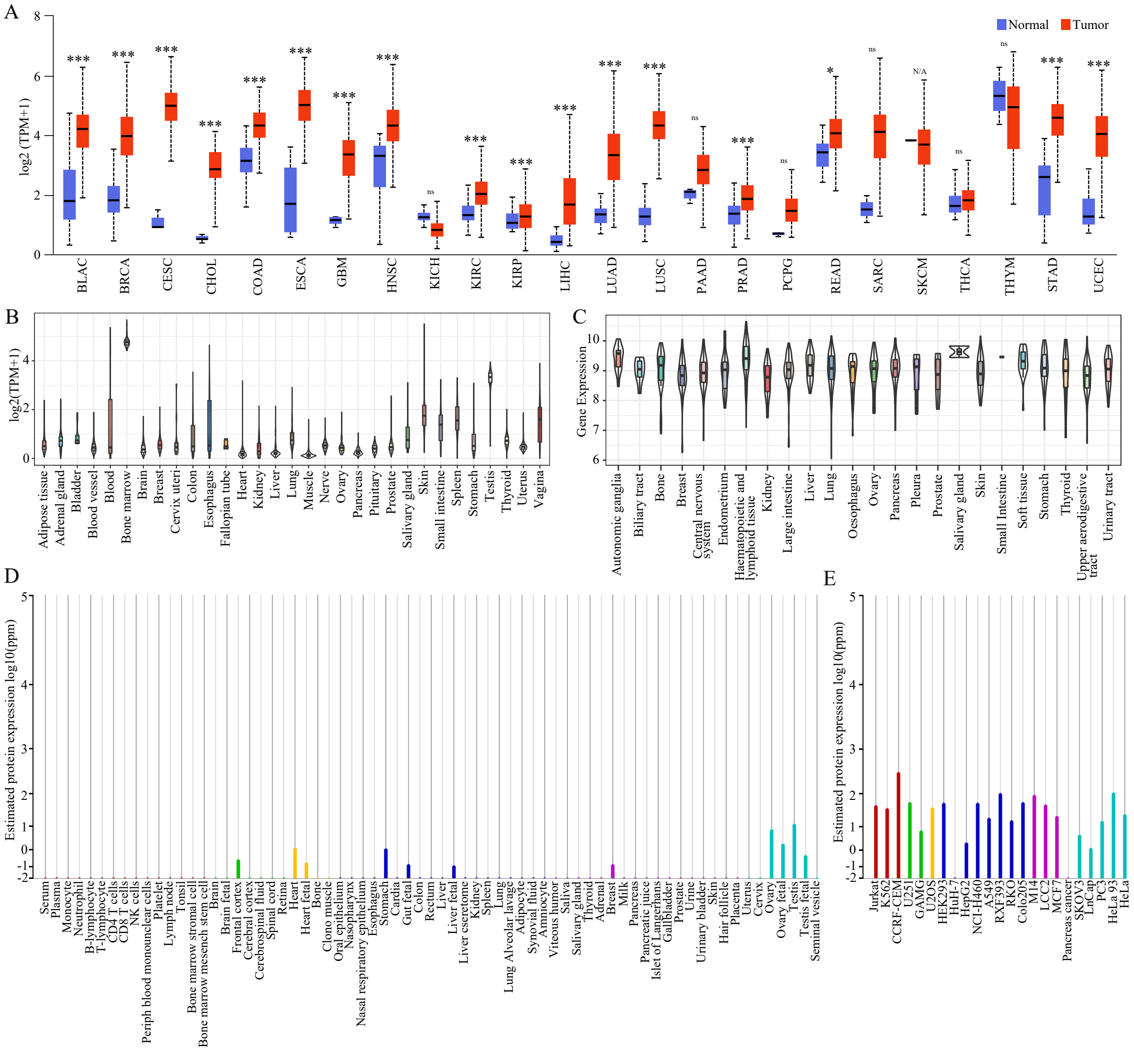
A
Normal
Tumor
log2 (TPM+1)
BLAC
BRCA
CESC
CHOL
COAD
ESCA
GBM
HNSC
KICH
KIRC
KIRP
LIHC
LUAD
LUSC
PAAD
PRAD
PCPG
READ
SARC
SKCM
THCA
THYM
STAD
UCEC
B
log2(TPM+1)
Adipose tissue
Adrenal gland
Bladder
Blood vessel
Blood
Bone marrow
Brain
Breast
Cervix uteri
Colon
Esophagus
Fallopian tube
Heart
Kidney
Liver
Lung
Muscle
Nerve
Ovary
Pancreas
Pituitary
Prostate
Salivary gland
Skin
Small intestine
Spleen
Stomach
Testis
Thyroid
Uterus
Vagina
C
Gene Expression
Autonomic ganglia
Biliary tract
Bone
Breast
Central nervous system
Endometrium
Haematopoietic and lymphoid tissue
Kidney
Large intestine
Liver
Lung
Oesophagus
Ovary
Pancreas
Pleura
Prostate
Salivary gland
Skin
Small Intestine
Soft tissue
Stomach
Thyroid
Upper aerodigestive tract
Urinary tract
D
Estimated protein expression log10(ppm)
E
Estimated protein expression log10(ppm)
Jurkat
K562
CCRF-CEM
U251
GAMG
U2OS
HEK293
HuH-7
HepG2
NCI-H460
A549
RXF393
RKO
Colo205
M14
LCC2
MCF7
Pancreas cancer
SKOV3
LnCap
PC3
HeLa 93
HeLa
